# Ecology and environment predict spatially stratified risk of H5 highly pathogenic avian influenza clade 2.3.4.4b in wild birds across Europe

**DOI:** 10.1038/s41598-025-30651-9

**Published:** 2025-12-02

**Authors:** Sarah Hayes, Joe Hilton, Joaquin Mould-Quevedo, Christl A. Donnelly, Matthew Baylis, Liam Brierley

**Affiliations:** 1 Department of Statistics, University of Oxford, Oxford, UK; 2 Pandemic Sciences Institute, University of Oxford, Oxford, UK; 3 Institute of Infection, Veterinary and Ecological Sciences, University of Liverpool, Liverpool, UK; 4 CSL Seqirus USA, Summit, NJ USA; 5 Institute of Population Health, University of Liverpool, Liverpool, UK; 6 Centre for Virus Research, MRC-University of Glasgow, University of Glasgow, Glasgow, UK

**Keywords:** Machine learning, Influenza virus, Biogeography, Ecological epidemiology

## Abstract

Highly pathogenic avian influenza (HPAI) represents a threat to animal and human health, with the ongoing H5N1 outbreak within the H5 2.3.4.4b clade being one of the largest on record. However, it remains unclear what factors have contributed to its intercontinental spread. We use Bayesian additive regression trees, a machine learning method designed for probabilistic modelling of complex nonlinear phenomena, to construct species distribution models (SDMs) for HPAI clade 2.3.4.4b presence. We identify factors driving geospatial patterns of infection and project risk distributions across Europe. Our models are time-stratified to capture both seasonal changes in risk and shifts in epidemiology associated with the succession of H5N6/H5N8 by H5N1 within the clade. While previous studies aimed to model HPAI presence from physical geography, we explicitly consider wild bird ecology by including estimates of bird species richness, abundance of specific taxa, and “abundance indices” describing total abundance of birds with high-risk behavioural traits. Our projections of HPAI clade 2.3.4.4b indicate a shift in persistent, year-round risk towards cold, low-lying regions of northwest Europe associated with H5N1. Methodologically, we demonstrate that while most variation in risk can be explained by climate and physical geography, adding host ecology is a valuable refinement to SDMs of HPAI.

**Supplementary Information:**

The online version contains supplementary material available at 10.1038/s41598-025-30651-9.

## Introduction

Avian influenza is a highly contagious viral disease caused by avian influenza type A viruses. It can have devastating effects on animal health and livestock economics with losses reaching $billions in the USA alone^1^, but also poses a threat to human public health and has pandemic potential. Spillover of avian influenza from birds to mammals is known to occur, but most spillover events are associated with little or no ongoing transmission. However, recent reports of mass mortality events in marine mammal populations in South America^2–4^ and farmed mink in Spain^5^ sparked concerns surrounding the potential for mammal-to-mammal transmission. The discovery of HPAI infection in cattle in North America in 2024^6,7^ and subsequent instances of onward transmission to farm workers^8^ have prompted high-priority surveillance responses considering the threat posed by potential human adaptation of the virus.

Whilst highly pathogenic avian influenza (HPAI) viruses may infect both domestic and wild birds, they are defined as those that cause severe disease in young poultry chicks intravenously inoculated in a laboratory setting, or those that possess multiple basic amino acids in the haemagglutinin cleavage site, which has been associated with high virulence. HPAI viruses can arise from changes to low pathogenic avian influenza (LPAI) viruses during circulation within poultry and are often detected from the resulting severe disease. HPAI is typically restricted to subtypes containing the H5 or H7 haemagglutinin glycoprotein (HA). Since 2002, symptomatic HPAI in wild birds has been increasingly recognised^9–13^. The H5 HPAI virus strain A/goose/Guangdong/1/1996 (H5N1) (gs/Gd) first emerged in geese in southeast Asia in 1996^14^ and was reported to cause wild bird mortality several years later^10^. By late 2005, gs/Gd H5 HPAI viruses were recognised in Europe, with the migration of wild birds implicated in this long-distance transmission^15,16^. Since then, genetic divergence and reassortment events have led to multiple European outbreaks of various clades within the gs/Gd H5 HPAI lineage in both domestic and wild birds^13,17^.

Since emerging out from the Qinghai Lake region, clade 2.3.4.4b has arisen as the dominant clade in Eurasia, with significant European outbreaks of H5N8 genotype viruses in 2016-17 and 2020–21^18–20^. A new reassortant H5N1 genotype then emerged in Europe around late 2020^19^, containing 2.3.4.4b HPAI HA and matrix protein (MP) genes, but LPAI-origin neuraminidase and internal genes^18^. This reassortant has since significantly expanded both in geography^17,21^ and host range^19^, affecting a much wider diversity of wild species, including gannets (Sulidae), pelicans (Pelicanidae), terns (Sterninae), and skuas (Stercorariidae).

Phylodynamic analyses also show wild birds experience longer duration of circulation, wider spatial dissemination, and greater likelihood of seeding infections of 2.3.4.4b viruses compared to domestic poultry^22^ or wild birds infected with previous H5Nx clades^18,19^, widening the possible palette of future reassortment risks in the wild. The apparent broader host susceptibility and persistence of H5N1 2.3.4.4b in particular is thought to be driven by further reassortment events with endemic gull H13 LPAI viruses^20^. Therefore, there is a critical need to better understand the changing role of wild birds in the transmission and maintenance of avian influenza viruses. 

There has been much interest in which species contribute to population maintenance of influenza viruses. In 2006, six HPAI H5N1 viruses were isolated from apparently healthy migratory ducks in China^23^ and recent infection studies have suggested that mallard ducks (*Anas platyrhynchos)* may shed H5N1 clade 2.3.4.4b virus whilst asymptomatic^24,25^. In addition to risks associated with particular host species, wider functional ecology of hosts has been cited as an important but neglected factor in the epidemic transmission of viral diseases of wild birds^26,27^. Behaviours that increase bird density and host-to-host direct and indirect contact rates such as colony breeding and pre-migratory congregation may be associated with increased risk of exposure^28^. Migration also places a high physiological demand on birds, which may disrupt immunity^29^ and thus increase susceptibility to infection, although evidence for this is somewhat conflicting^28^. For LPAI, viruses in water can remain infectious for more than 30 days at 0 °C with spread predominantly via the faecal-oral route therefore dependent on foraging behaviours. The high prevalence of LPAI viruses in dabbling ducks may be in part due to exposure via surface water feeding^30,31^. However, HPAI viral shedding has also been linked to the respiratory route^32–35^ which may alter dependency of transmission on foraging behaviours. Even further unknown is the specific ecology of H5 HPAI clade 2.3.4.4b viruses and whether it may explain their apparent diversity of susceptible hosts.

Species distribution models (SDMs) make predictions across geographic space and time using a set of predictor variables. They have traditionally been used in ecology to predict the distribution of plant and animal species using environmental variables, but more recently have been applied to infectious disease^36–40^. SDMs have been used for modelling avian influenza viruses in domestic birds^41–47^, wild birds^48–54^, and both combined^55,56^. Models have been produced at local^52–54^ and continental resolutions^48–51^ but few have adopted more recent approaches to integrate host ecology at large spatial scales^26^, instead using only environmental predictors. Notable exceptions include Belkhiria et al.^52^, who included Important Bird and Biodiversity Areas as model predictors to act as a proxy for the presence of migratory birds, whilst Moriguchi et al.^53^ included population sizes of migratory and dabbling ducks. Hill et al.^57^ used poultry holding densities, poultry species-specific risk estimates, and wild bird population sizes to inform a spatial mechanistic model of incursion into poultry farms in the UK, but did not include physical geography in their model. Huang et al.^50^ explored more sophisticated ecological measures of host community composition on HPAI H5N1 across Europe, incorporating features such as species richness and abundance of waterbirds in general and of specific waterbird high-risk hosts, as well as phylogenetic relatedness to those high-risk hosts. However, none of these previous studies combine a continental scale of analysis, ecological traits of the host, and a direct comparison between the current outbreak of HPAI H5N1 with that of previously circulating H5 HPAI lineages within clade 2.3.4.4b.

The aim of this study is to map and understand the geospatial risk of H5 HPAI presence across Europe using an SDM approach. Our modelling builds on previous studies which focus on physical geography by explicitly incorporating ecological covariates including avian species richness, abundance of avian species with specific behavioural risk factors, and abundance of specific high-risk avian taxa. We train a Bayesian additive regression trees (BART) model on H5 HPAI detections of clade 2.3.4.4b since 2016 and use data on the physical geography and ecology of over 600 wild bird species of Europe to estimate the probability of H5 HPAI clade 2.3.4.4b presence at a 10 km resolution. Although we primarily use models to identify specific locations at high risk of acting as epicentres for future HPAI outbreaks, we also calculate variable importance and partial dependence to interrogate which specific risk factors are highly predictive of different subtypes of H5 clade 2.3.4.4b and why, including novel contributions from host ecological data. We sense-check our models by comparing their risk projections with data on domestic bird H5 HPAI detections, as introduction of H5 HPAI into domestic poultry may result from contact with infected wild birds or wild bird-derived fomites, and so we expect domestic poultry cases to be more likely to occur in areas where we predict high risk of H5 HPAI in wild birds.

## Methods

**Table 1:** Environmental covariates included in the models.

Topographic covariates	Details	Resolution of data source	Year of data	Source
Elevation	Minimum elevation (metres above sea level)	30 arc seconds	GTOP030–1999 CGIAR-SRTM − 2018	Geodata package R https://cran.r-project.org/web/packages/geodata/geodata.pdf
Elevation	Maximum elevation (metres above sea level)	30 arc seconds	GTOP030–1999 CGIAR-SRTM − 2018	Geodata package R https://cran.r-project.org/web/packages/geodata/geodata.pdf
Elevation	Difference between minimum and maximum elevation (m)	30 arc seconds	GTOP030–1999 CGIAR-SRTM − 2018	Processed using minimum and maximum elevation listed above
Elevation	Modal elevation (metres above sea level)	30 arc seconds	GTOP030–1999 CGIAR-SRTM − 2018	Geodata package R https://cran.r-project.org/web/packages/geodata/geodata.pdf
Normalised difference vegetation index (NDVI)	Mean over the season. Normalised to limit values to between − 1 and 1	1 km	2022	Modis https://modis.gsfc.nasa.gov/data/dataprod/mod13.php
Land cover	One raster layer for each of the 17 land cover categories	0.5 km	2022	Modis https://lpdaac.usgs.gov/products/mcd12q1v061/
Distance to coast	Distance from centre of raster cell to nearest coast (km)		N/A	Calculated directly from map data
Distance to inland water	Distance from centre of raster cell to nearest permanent inland water (m)	30 arc seconds	2004	Calculated using data from Global Lakes and Wetland Database https://www.worldwildlife.org/publications/global-lakes-and-wetlands-database-lakes-and-wetlands-grid-level-3
Bioclimatic covariates				
Relative humidity	Seasonal mean of daily midday (local time) relative humidity measured at 2 m (%)	0.1 degrees	2022	Copernicus https://cds.climate.copernicus.eu/cdsapp#!/dataset/sis-agrometeorological-indicators? tab=overview
Temperature	Seasonal weighted mean of the month-wise difference in the minimum temperature and maximum temperature (degrees Celsius)	2.5 arc minutes	2018	WorldClim https://www.worldclim.org/data/monthlywth.html
Temperature	Seasonal weighted mean of monthly mean temperatures (degrees Celsius) (Mean monthly temperature for each month calculated using: Mean temperature = Minimum temperature + diurnal range/2)	2.5 arc minutes	2018	WorldClim https://www.worldclim.org/data/monthlywth.html
Temperature	Seasonal temperature variation (degrees Celsius) (Difference between the maximum and minimum of mean monthly temperature values across months majority-represented within the season)	2.5 arc minutes	2018	WorldClim https://www.worldclim.org/data/monthlywth.html
Precipitation	Seasonal weighted mean of the monthly total precipitation (mm)	2.5 arc minutes	2018	WorldClim https://www.worldclim.org/data/monthlywth.html
Zero-degree isotherm	Seasonal mean of daily zero-degree isotherm (metres above sea level)	0.25 degrees	2022	Copernicus https://cds.climate.copernicus.eu/cdsapp#!/dataset/reanalysis-era5-single-levels? tab=overview
Zero-degree isotherm	Number of days the zero-degree isotherm was below 1 m at midday at Coordinated Universal Time (UTC)	0.25 degrees	2022	Copernicus https://cds.climate.copernicus.eu/cdsapp#!/dataset/reanalysis-era5-single-levels? tab=overview
Livestock demography				
Chicken density	Headcount of animals	5 arc minutes	2010	Gridded livestock of the world 3 https://www.fao.org/livestock-systems/global-distributions/chickens/en/
Duck density	Headcount of animals	5 arc minutes	2010	Gridded livestock of the world 3 https://www.fao.org/livestock-systems/global-distributions/ducks/en/

### Data assembly and season definition

We assembled a set of geospatial covariate rasters at a 10 km spatial resolution. This resolution was chosen to smooth potential error in geolocated coordinates of positive H5 HPAI records given the wide-ranging movement of wild bird hosts, and for close alignment with the native resolution of many covariates (Table 1). We used the EPSG:3035 coordinate reference system, which is intended for spatial statistical analysis across continental Europe^58^. To account for seasonal changes in risk, we assembled data for four seasonal periods as an overall calendar proxy for European bird behavioural cycles: non-breeding season, 30th November − 28th (or 29th) February (calendar days 334 − 59); pre-breeding migration, 1 st March − 6th June (days 60–157); breeding season, 7th June − 9th August (days 158–221); and post-breeding migration, 10th August − 29th November (days 222–333).

These periods were chosen by querying seasonal status for each bird species listed as present in Europe within the 2022 data release of eBird Status and Trends^59^. These data describe numerical calendar weeks of each individual bird species’ life cycle season (“breeding”, “nonbreeding”, “post-breeding migration”, and “pre-breeding migration” seasons, or “resident” for non-migratory species). We defined aggregated proxy behavioural seasons such that a plurality of migratory bird species are in the given season at the given time (Supplemental Methods S1, Supplemental Fig. S1A). For example, from 30th November − 28th February, the majority seasonal status among migratory birds is ‘non-breeding’. At the species level, our behavioural seasons are intended to capture the modal behaviour of European seasonal birds.

### Environmental covariates

We selected a set of environmental covariates consistent with those reported to be important in avian influenza dynamics^48–54,60^. Covariates were categorised as topographical, bioclimatic or livestock demography and are listed in Table 1. There was a large amount of variability in the resolution and coordinate reference systems of the different data sets used. Where calculations were required these were done at the original resolution prior to reprojection. Rasters were then reprojected to the EPSG:3035 coordinate reference system and the resolution converted to 10 km using bilinear interpolation for continuous covariates and nearest neighbour for categorical covariates (Supplemental Figure S2).

#### Topographical

Elevation data were sourced using the R package “geodata” v0.5–8^61^ which combines Shuttle Radar Topography Mission (SRTM) data and Global 30 Arc-Second Elevation (GTOPO30) data. Elevation data were processed to extract the minimum, maximum and modal elevation for each of the 10 km resolution cells of our project area. A fourth elevation raster containing the difference between the minimum and maximum values recorded within each of the cells was also produced.

The normalised difference vegetation index (NDVI) is a measure of the vegetation canopy greenness and acts as a marker of vegetation density. NDVI data were sourced from NASA’s Moderate Resolution Imaging Spectroradiometer (MODIS) data in 16-day periods at a 1 km resolution. The data were grouped to reflect the seasons as closely as possible by assigning each 16-day period to the season the majority of its days fell within. A mean NDVI for each aggregated bird behavioural season was calculated and standardised to limit the range between − 1 and 1.

Data on land cover were sourced from MODIS Land Cover Type (MCD12Q1) Version 6.1. Land cover was categorised as one of 17 different land cover types numbered 1–17. During reprojection, the nearest neighbour method was used to create a raster at 10 km resolution. Separate rasters for each of the land cover types are then created with the value of the cell representing whether or not that land cover type is assigned to that cell (Supplemental Fig. S2).

Inland water bodies were identified using the Global Lakes and Wetlands database provided by the World Wildlife Fund. We excluded intermittent (i.e., non-permanent) lakes and wetlands with the resulting raster layer containing distance to any permanent water body of any type.

#### Bioclimatic

Temperature and precipitation data were obtained from WorldClim historical monthly data for the most recent year available at initial download (2018) at a 2.5 arc minute resolution. Temperature data are entered as seasonal weighted mean of monthly mean temperatures (weighting by number of days of each month represented in the behavioural season), short-term variability in temperature taken as weighted mean of the month-wise differences in minimum and maximum temperatures, and the overall variation in mean temperature over the season (calculated by taking the difference between the minimum and the maximum of the mean temperatures across all months with at least half their days represented in the respective season). Precipitation is entered as the mean (weighted by days falling in season, as for temperature) of the monthly values for total precipitation for each of the months of the season (Supplemental Fig. S2).

Relative humidity data were downloaded from the European Centre for Medium Range Weather Forecasts (ECMWF) Copernicus dataset, obtained by the ERA-Interim reanalysis of monthly observations from 1979 to the present. Data reflect the daily relative humidity at 2 m above ground level at midday local time. Mean seasonal humidity was calculated as the mean of the daily humidity values within the season.

Daily zero-degree isotherm data were also obtained from Copernicus and reflect the height above the Earth’s surface where the temperature in Celsius passes from positive to negative values at the specified time. Seasonal mean zero-degree isotherm and the number of days within the season where the zero-degree isotherm was below 1 m at midday (UTC) are included in the model (Supplemental Fig. S2).

#### Livestock demography

Data regarding the density of chickens and ducks were obtained from Gridded Livestock of the World version 3 (Supplemental Figure S2). Version 3 was used for both species due to large amounts of missing data for ducks in version 4.

**Table 2:** Ecological covariates included in the models.

Covariate	Total abundance or behaviour-weighted?	Species-level source
Anatinae (“dabbling ducks”)	Total	eBird https://science.ebird.org/en/status-and-trends
Anserinae (swans and geese)	Total	eBird https://science.ebird.org/en/status-and-trends
Ardeidae (herons)	Total	eBird https://science.ebird.org/en/status-and-trends
*Arenaria/Calidris* (turnstones and sandpipers)	Total	eBird https://science.ebird.org/en/status-and-trends
Aythyini (“diving ducks”, or pochards)	Total	eBird https://science.ebird.org/en/status-and-trends
Laridae (gulls)	Total	eBird https://science.ebird.org/en/status-and-trends
Percentage time spent feeding within 2 m of water surface	Weighted	EltonTraits 10.6084/m9.figshare.c.3306933.v1
Percentage time spent feeding > 2 m below water surface	Weighted	EltonTraits 10.6084/m9.figshare.c.3306933.v1
Percentage diet plants	Weighted	EltonTraits 10.6084/m9.figshare.c.3306933.v1
Percentage diet scavenging	Weighted	EltonTraits 10.6084/m9.figshare.c.3306933.v1
Percentage diet endothermic vertebrates	Weighted	EltonTraits 10.6084/m9.figshare.c.3306933.v1
Congregative	Total	IUCN Red List https://www.iucnredlist.org/
Migratory	Total	IUCN Red List https://www.iucnredlist.org/
Below threshold phylogenetic distance to known host species	Total	BirdTree https://birdtree.org/ CLOVER https://github.com/viralemergence/clover
Species richness	N/A	eBird https://science.ebird.org/en/status-and-trends

### Ecological covariates

We constructed raster layers of ecological risk factors by combining species-level risk factors, estimates of global species population and relative spatial abundance data into a single index of ‘species-trait abundance’ (Supplemental Figure S3). To reflect the key role of migratory behaviour in wild bird H5 HPAI epidemiology^22,27,28,62^ we obtained binary species-level indicators specifying whether a given species was migratory (defined as having ‘Full Migrant’ classification) and/or congregative from the IUCN Red List^63^. To capture bird diet and foraging behaviour, we took estimates from EltonTraits^64^ of the percentage contribution of specific food sources and foraging behaviours to each species’ total diet and time spent foraging. Of the food sources considered in EltonTraits, we considered plants and endothermic vertebrates (mammals and birds). Of the foraging behaviours, we considered feeding within 2 m of water surfaces, feeding below the water surface at depths exceeding 2 m, and scavenging. Evolutionary relatedness between hosts is a well-established predictor of infection potential in various systems^65,66^, including avian influenza^67^. To capture this effect of phylogenetic structure in susceptibility across avian host species, we downloaded 100 posterior estimates of the bird phylogenetic tree from BirdTree^68^ and used the function cophenetic.phylo from the R package ape, v5.8^69^ to calculate a matrix of pairwise phylogenetic distances between bird species. We used the CLOVER database^70^ to identify known avian influenza host species, and for each species in the BirdTree phylogeny calculated the minimum phylogenetic distance to a known host species. We converted this to an indicator variable specifying for each species whether the phylogenetic distance to any known avian influenza host species met a threshold value of 50 million years since lineage divergence. This was chosen by inspecting fitted functions from a preliminary model trained to predict host species status, where 85% of known host species fell within this threshold distance from the nearest heterospecific host species. Taxonomic conventions differed between data sources, with disagreements over both species names and distinctions between species and subspecies. To allow for cross-referencing between data sources with differing taxonomic conventions we performed a semi-automated rationalisation process using the AVONET database of bird species names^71^, detailed in Supplemental Methods S1. A complete list of ecological covariates used in our models is given in Table 2.

We obtained estimates of species abundance in the form of percentage population at a 9 km grid resolution from the spatiotemporal component of eBird Status and Trends. These estimates are calculated using species distribution models trained on checklist-based bird observation data submitted to the eBird citizen science platform, and, for a given species, take the form of 52 weekly geospatial raster layers specifying the percentage of the global population of a given species in each grid cell in each week of the year. The species modelled by eBird include only a subset of those known to be present in Europe; a total of 708 species as opposed to 944 species listed as being observed in Europe in Avibase^72^. For each species in each behavioural season, eBird provides quality ratings on a scale of 0 (lowest quality) to 3 (highest quality).

We used the species-specific quality metrics and temporal boundaries to generate weekly quality measures, and for each aggregated behavioural season included only those species which had a quality rating ≥ 2 for the majority of the season. We defined the estimated abundance of a given species in a given grid cell for a given aggregated behavioural season to be the mean of the weekly abundance across all weeks in that season with quality rating ≥ 2. To convert from percentage population to absolute abundance we subsequently scaled each species’ abundance raster by the corresponding estimated global population size provided by Callaghan et al.^73^, giving us an estimated absolute abundance raster (Supplemental Figure S3). After discarding species with problematic taxonomic statuses and year-round low quality levels we were left with 617 of the 708 European species modelled by eBird Status and Trends.

We used these estimated absolute abundance layers to develop spatial raster layers representing indices of population-level ecological traits for use as covariates (Supplemental Figure S3). Species richness was estimated by counting the total number of species in each grid cell that had a total estimated abundance greater than 1. For migratory behaviour, congregative behaviour, and phylogenetic host distance threshold, we created indices by adding together all the abundance layers for species which had a positive indicator for these factors. For the dietary and foraging behaviour risk factors, we summed all of the abundance layers weighted by the percentage of dietary and foraging behaviour allocated to the specific behaviour for each species in EltonTraits, to give a measure of relative population behaviour in each grid cell. We constructed covariate layers representing six specific bird taxa by adding together all the abundance layers for species in each. These taxa were families Anatinae, Anserinae, Ardeidae, Aythyini, Laridae, as well as genera *Arenaria* and *Calidris*, which were combined in a single layer due to their close relatedness and the small number of species in *Calidris*^68^. These taxa were chosen based on high numbers of appearances in the CLOVER database^70^ or previous identification as high-risk avian influenza species^74^.

### Outcome data

Data on confirmed avian influenza cases in wild birds were obtained from publicly available data sources: The Food and Agricultural Organization of the United Nations’ (FAO) EMPRES-i and the World Animal Health Information System (WAHIS) database provided by the World Organization for Animal Health (WOAH). Data spanned from August 2016 until February 2024 and were filtered to H5 HPAI within the specified study area. Duplicate entries based on date and geographical coordinates were removed. Data entries that had the same date and geographical coordinates but were listed as different subtypes were retained within the data. Data were mapped to a 10 km grid considering cells positive if they contained ≥ 1 H5 HPAI record.

**Fig. 1:**
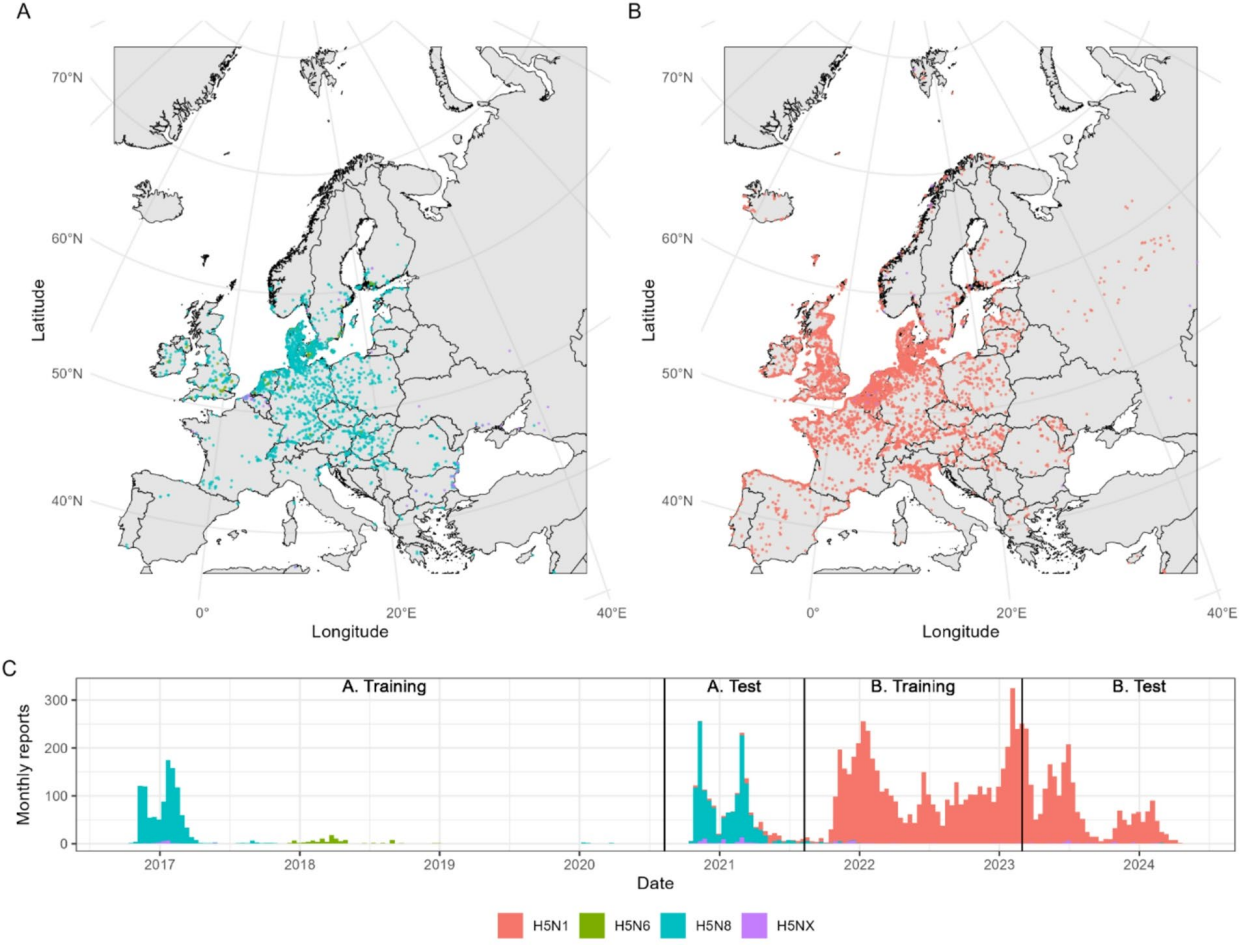
Wild bird H5 HPAI clade 2.3.4.4b incident data in Europe. Spatiotemporal distribution of incidents of H5 highly pathogenic avian influenza clade 2.3.4.4b reported in wild birds, harmonised from EMPRES-i and WAHIS databases and before assignment to 10 km spatial grids. (**A**) and (**B**) Geolocated incident reports over time periods (**A**) (10/8/2016–9/8/2021) and (**B**) (10/8/2021–29/2/2024), respectively. (**C**) Total incident reports per month with separate model training and test periods annotated. Incident reports depicted are independent geolocated instances of authority-reported positive H5 HPAI results, regardless of number of bird species or individuals involved. Colour denotes subtype recorded (H5N1; H5N6; H5N8; or other H5 where full subtype not explicitly labelled, denoted H5NX). Country boundary data are sourced from EuroGeographics and UN-FAO open geodata (https://ec.europa.eu/eurostat/web/gisco/geodata/administrative-units/countries) before reprojection to EPSG:3035 co-ordinate system. Plots generated with R package “ggplot2”, v3.5.1 (https://ggplot2.tidyverse.org).

Based on patterns of epidemic peaks and subtype emergence in Europe (Fig. 1), independent models were constructed to capture (A) discrete H5 HPAI outbreaks within clade 2.3.4.4b of H5N8 and H5N6 from 2016 to 2021 and (B) the extended H5N1 clade 2.3.4.4b epizootic, ongoing since 2021. Based on behavioural season boundaries, each set was divided into a training period, and a subsequent test period held out from training to assess model performance in near-future projections. For (A) models were trained on cases from 10/8/2016 to 9/8/2020, capturing H5N8 and H5N6 events (Fig. 1), before testing on a separate H5N8 outbreak from 10/8/2020 to 9/8/2021. For (B) models were trained on H5N1 cases from 10/8/2021 to 28/2/2023 before testing on H5N1 cases from 1/3/2023 to 29/2/2024. Training/test divisions were chosen to cover a minimum of one full bird behavioural season of each type (Supplemental Fig. S1B).

### Pseudo-absence generation

SDM analyses are often challenged by lack of available absence data (i.e., true negatives) to train on alongside presences; for HPAI, few authorities systematically report their negative test results. Several approaches exist to handle this problem, including presence-only or ‘one-class’ classification and niche boundary estimation^75,76^. We use an established solution in generating ‘pseudo-absence’ or background points, that can fulfil the role of negative training data by representing areas the modelled organism or disease could feasibly be present in but has never been observed in. It is strongly emphasised that pseudo-absences should be drawn to reflect the same biological restrictions and spatial sampling heterogeneities as presences^75,77^. We expect influenza surveillance in wild birds to be restricted by both human population to report potential cases and physical accessibility to conduct sampling. Therefore, to approximate these processes, we obtained general citizen surveillance records of wild bird sightings.

We obtained all bird observations made by the public in eBird by downloading the eBird Basic Dataset (EBD)^78^ before filtering to records within Europe using R package `auk`, v0.7.0^79^. We then calculated total sightings (i.e., unique data-user-geolocation records, regardless of species sighted) per 10 km grid cell over the study period 10/8/2016–29/2/2024 to create a spatial raster layer approximating bird sampling accessibility (Supplemental Figure S4). Pseudo-absences were then sampled from all cells that remained H5 HPAI-free during the entire study period and were ≥ 25 km from the nearest positive cell. Pseudo-absences were drawn at an initial 1:1 ratio with positives for each season using function add_pseudoabsence() from R package `ibis.iSDM` v0.1.2^80^, weighting by normalised total eBird sightings. We therefore preferentially select cells with high human density and sampling accessibility yet have never reported H5 HPAI as those we have stronger confidence in to represent true negatives. Pseudo-absences were generated jointly for training/test sets to avoid positive cells from one being considered pseudo-absences in the other.

### Data resampling

As a result of sampling patterns, spatial occurrence records for organisms or diseases are commonly concentrated in clusters, which can bias SDMs and their predictions. As in other computational fields, these biases can be mitigated by resampling training data with spatial data thinning being a popular approach^81^. However, at continental scales this may retain records that are geographically diverse, but capture little environmental variation. 

Therefore, we reduce training data redundancy by environmental thinning, which typically outperforms spatial thinning^82,83^. Considering environmental traits as an n-dimensional space and dividing each trait into a given number of bins, simple random stratified sampling can be conducted based on environmental uniqueness^82^, i.e., if there are many positive sites having characteristics falling within given ranges of temperature, humidity, precipitation, etc., only one needs to be retained to represent this niche. We thinned using the function `occfilt_env()` from R package `flexsdm`, v1.3.4^84^, based on nine purely environmental covariates (mean relative humidity, mean temperature, temperature range, temperature variation, total rainfall, max elevation, NDVI, distance to coast, distance to inland water), each stratified into six bins. Thinning was conducted independently for positive cells and pseudo-absence cells from each season’s training data from each dataset, resulting in between 188 and 1631 total cells (Table 3), excluding breeding season for period A which was not modelled due to lack of sufficient data.

**Table 3:** Data availability for model training and test sets in terms of number of grid cells. Value denotes number of positive grid cells, and bracketed value denotes number of grid cells sampled as pseudo-absences.

Dataset	Season	Raw training	Thinned training	Test
A	Nonbreeding (Nov - Feb)	589 (548)	408 (460)	453 (422)
	Pre-breeding migration (Mar - Jun)	107 (104)	89 (99)	336 (325)
	Breeding (Jun - Aug)	7 (7)	5 (7)	21 (21)
	Post-breeding migration (Aug - Nov)	183 (177)	131 (166)	288 (279)
B	Nonbreeding (Nov - Feb)	1595 (1380)	635 (966)	326 (282)
	Pre-breeding migration (Mar - Jun)	415 (384)	218 (330)	754 (698)
	Breeding (Jun - Aug)	360 (339)	142 (302)	488 (460)
	Post-breeding migration (Aug - Nov)	957 (885)	380 (669)	188 (174)

### Modelling approach

Using the above training data, we fitted BART, an ensemble-based nonparametric machine learning method based around constructing large numbers of decision trees^85^. The decision tree structure of BART makes it ideal for modelling complex nonlinear relationships such as environment-host-disease interactions. An ensemble of candidate models is constructed via Markov Chain Monte Carlo (MCMC), with the predicted response value for a given covariate datapoint given by the mean predicted response across these candidate models. One particular advantage of BART is that credible intervals can be generated by taking quantiles of the ensemble of projected values. We carried out our model fitting using the R programming language package *embarcadero*^86^, which offers an implementation of BART designed to interface with geospatial data and generate spatial projections; based on a fitted BART model *embarcadero* can generate a raster of predicted response variables from a multilayer raster of covariates.

We explore a basic model, plus a model including cross-seasonal covariates. In the basic model, we construct models using seasonally-variable environmental and ecological covariates for only the same behavioural season as the H5 HPAI records being trained on, whereas with cross-seasonal covariates, we construct using seasonally-variable covariates from all four seasons. This is to allow for potential delay effects in the infectious disease dynamics; for instance, winter case numbers in a given location could be influenced by the local climate over summer, or large numbers of high-risk birds congregating in a location at one time of year could drive the circulation of infection lasting throughout the year. We also test a model formulation specifying country-level random intercepts (Supplemental Methods S1).

We performed five-fold cross validation to optimise the parameters of the MCMC, and performed stepwise variable set reduction to optimise our covariate set for predictive ability. We fit a separate instance of BART for each of our seasonal datasets, with the variable set reduction allowing models for different seasons to retain distinct covariates, reflecting annual variation in epidemiology. Uniform priors were specified for the probability of each covariate being selected at a given tree branching point, and on the exact split point of the covariate used in branching. A negative power law prior was specified for the probability of incrementing tree depth $\:d$ as $\:{\alpha\:(1+d)}^{-\beta\:}$ where $\:a$ was set to 2 and $\:\beta\:$ was set to 0.95 following package defaults^86^. We ran eight parallel MCMC chains of length 1000 plus a burn-in period of length 100, generating a total of 8000 posterior samples for each model instance.

### Comparison to domestic cases

We sense-checked our predictions by calculating the odds of observing a domestic bird H5 HPAI detection in locations where domestic birds were present, stratified by model-projected risk. We extracted data on domestic bird detections of HPAI with a known H5 subtype from the same sources and over the same period as for wild bird cases. To ensure we only looked at cells with non-negligible domestic bird populations we added together chicken and domestic duck density raster layers to produce a raster capturing the combined density of both species. We first selected all 10 km grid cells with > 1000 domestic ducks and chickens combined and labelled them as infected or uninfected based on the presence of at least one reported domestic case of H5 HPAI. Each cell was also associated with a model-predicted probability of H5 HPAI presence in wild birds. For each 10% band of predicted probability, we calculated the risk-stratified odds of infection as the number of cells with domestic bird H5 HPAI detection divided by the number of cells without (each restricted to those with > 1000 domestic ducks and chickens combined). For each period (A and B) and for each season this gave a set of 10 risk-stratified odds which were visualised to examine potential linkage between H5 HPAI outbreaks in wild and domestic birds.

The code used to generate our training data and perform our analysis is available at https://github.com/sarahhayes/avian_flu_sdm.

## Results

Based on performance metrics in predicting held-out test data (Table 4), our BART models trained on environmental and ecological predictors showed good generalisability in understanding the distribution of H5 HPAI clade 2.3.4.4b for each modelled season in both data periods (Table 4). Specificity and sensitivity were generally well-balanced, though for the ongoing (since 2021) H5N1 outbreak performance in warmer periods (pre-breeding migration and breeding seasons) was slightly weaker in sensitivity and overall Area Under Receiver-Operating Characteristic curve (AUROC), likely due to much smaller training data availability compared to test set for these seasons (Table 3; Supplemental Fig. S1B). Including a random intercept term stratified by country did not noticeably improve overall AUROC for either dataset and reduced sensitivity in some cases (Supplemental Methods S1; Supplemental Table S1). Allowing models to access cross-seasonal covariates had surprisingly minimal impact upon model performance (Table 4), though we retain these as our preferred model suite to allow us to investigate ecological impacts of annual migration cycles on year-round avian influenza risk.

**Fig. 2:**
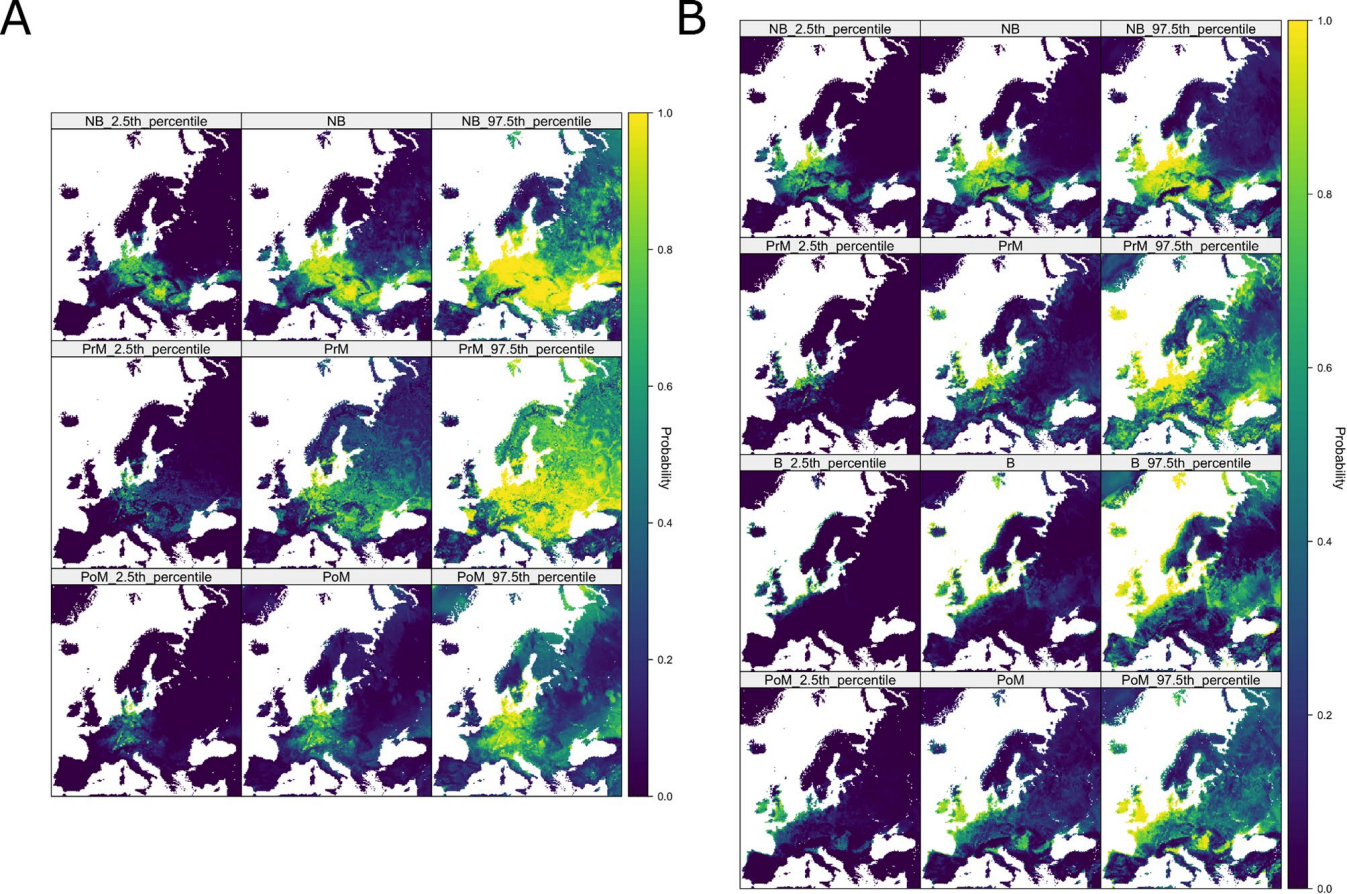
Projected risk maps for H5 HPAI clade 2.3.4.4b and associated uncertainty intervals. Projected probability of H5 HPAI clade 2.3.4.4b presence by aggregated bird behavioural season (NB = Nonbreeding season, PrM = Pre-breeding migration, B = Breeding season, PoM = Post-breeding migration) during period (**A**) (10/8/2016 to 9/8/2021, spanning H5N8 and H5N6 events) and period (**B**) (10/8/2021 to 29/2/2024, ongoing H5N1 outbreak) from selected final models with cross-seasonal covariates. Central columns are posterior means from BART ensemble, left and right columns represent 95% credible intervals.

We generated 10 km resolution risk projections for H5 HPAI clade 2.3.4.4b across Europe with 95% credible intervals (Fig. 2). Considering discrete outbreaks of H5N6 and H5N8 clade 2.3.4.4b (period A), risk hotspots for H5 HPAI in wild birds were present across central Europe during the aggregated non-breeding season, dissipating northward during pre-breeding migration (Fig. 2A). While the breeding period lacked sufficient data to model, we note a more limited risk distribution during post-breeding migration (Fig. 2A). Restricting to highest-confidence hotspots of H5 HPAI presence (high risk at 2.5th percentiles) showed consistent risk concentrated around central and eastern European shorelines and waterways, while restricting to highest confidence hotspots of H5 HPAI absence (low risk at 97.5th percentiles) suggested low suitability for H5 HPAI in high-elevation, mountainous regions as well as the Iberian peninsula.

Contrastingly, we found the ongoing H5N1 epizootic in wild birds (period B) to have much more spatially concentrated risk (Fig. 2B). Predicted H5N1 HPAI hotspots appeared further westward and centred around the British Isles and North Sea coast with comparatively lower risk throughout eastern Europe (Fig. 2B). Presence of H5N1 also appeared less seasonally-variable than for H5N6 and H5N8, with only minimal northward shift during pre-breeding migration (Fig. 2B), though risk during the breeding season was highly localised to north-western European coastlines. Although different environments were observed to be at higher risk compared to previous outbreaks, the lowest risk was again consistently observed for more mountainous and arid regions.

**Table 4:** Posterior means of performance metrics for each model option on each dataset’s test set. AUROC denotes area under the Receiver-Operating characteristic curve, CV denotes covariates. Presence data in test set A is from the 2020–2021 H5N8 HPAI clade 2.3.4.4b outbreak. Presence data in test set B is from 1/3/2023 to 29/2/2024, during the ongoing (since 2021) H5N1 HPAI clade 2.3.4.4b outbreak. Values in brackets denote posterior median and 95% credible interval of metrics within individual trees. As posterior mean values are based on consensus predictions averaging over all sampled trees, this can exceed posterior medians and 95% credible intervals.

			Model	
			Base model	+ Cross-seasonal CV’s
A	Nonbreeding (Nov - Feb)	Sens. Spec. AUROC	0.77 (0.76; 0.72–0.81) 0.83 (0.82; 0.77–0.87) 0.90 (0.88; 0.85–0.90)	0.80 (0.80; 0.76–0.84) 0.81 (0.80; 0.75–0.85) 0.90 (0.88; 0.86–0.90)
	Pre-breeding migration (Mar - Jun)	Sens. Spec. AUROC	0.77 (0.77; 0.69–0.83) 0.89 (0.83; 0.74–0.90) 0.91 (0.88; 0.84–0.90)	0.86 (0.83; 0.76–0.88) 0.83 (0.77; 0.65–0.86) 0.92 (0.89; 0.85–0.91)
	Breeding (Jun - Aug)	Sens. Spec. AUROC	-	-
	Post-breeding migration (Aug - Nov)	Sens. Spec. AUROC	0.88 (0.86; 0.77–0.91) 0.79 (0.76; 0.64–0.83) 0.86 (0.85; 0.80–0.90)	0.86 (0.86; 0.80–0.91) 0.75 (0.74; 0.68–0.78) 0.86 (0.85; 0.81–0.89)
B	Nonbreeding (Nov - Feb)	Sens. Spec. AUROC	0.83 (0.81; 0.77–0.84) 0.84 (0.84; 0.80–0.90) 0.93 (0.91; 0.89–0.93)	0.80 (0.80; 0.77–0.83) 0.83 (0.83; 0.80–0.87) 0.91 (0.90; 0.88–0.92)
	Pre-breeding migration (Mar - Jun)	Sens. Spec. AUROC	0.80 (0.78; 0.72–0.84) 0.61 (0.61; 0.53–0.69) 0.79 (0.75; 0.71–0.79)	0.77 (0.75; 0.70–0.80) 0.69 (0.64; 0.56–0.71) 0.79 (0.75; 0.71–0.79)
	Breeding (Jun - Aug)	Sens. Spec. AUROC	0.84 (0.81; 0.76–0.85) 0.65 (0.62; 0.56–0.68) 0.79 (0.77; 0.74–0.80)	0.83 (0.81; 0.77–0.84) 0.64 (0.62; 0.57–0.67) 0.76 (0.76; 0.72–0.79)
	Post-breeding migration (Aug - Nov)	Sens. Spec. AUROC	0.83 (0.80; 0.75–0.85) 0.84 (0.78; 0.67–0.84) 0.88 (0.86; 0.81–0.88)	0.80 (0.77; 0.72–0.83) 0.85 (0.83; 0.77–0.87) 0.89 (0.87; 0.85–0.89)

**Fig. 3:**
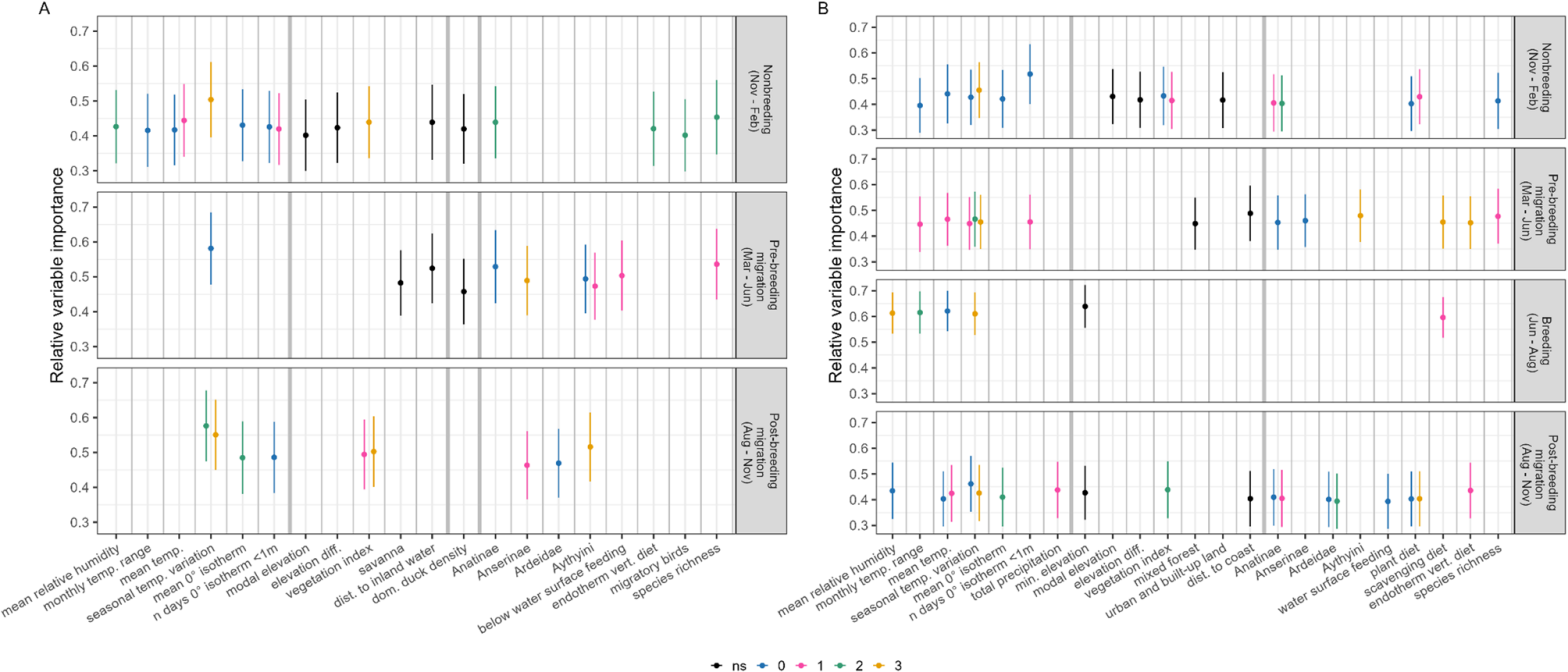
Variable importance of BART models. Relative variable importance of each covariate kept in optimal fitted BART models for period (**A**) (10/8/2016 to 9/8/2021, spanning H5N8 and H5N6 events) and period (**B**) (10/8/2021 to 29/2/2024, ongoing H5N1 outbreak). Points denote normalised proportion of times variable was used in a tree decision split out of all splits, averaged over 8000 draws from the posterior tree space. Error bars denote +/- 1 SD. Black denotes non-seasonally-variable covariates (“ns”), while colour denotes seasonally-variable covariates. Exact colour key indicates lag (precedence) of covariate season to modelled season, e.g., for the breeding season model, blue points represent covariate values from the breeding season itself (lag 0); pink points represent covariate values from one season prior (lag 1), i.e., non-breeding migration; green points represent covariate values from two seasons prior (lag 2), i.e., nonbreeding season; etc. Thicker grey lines separate bioclimatic, topographic, livestock demography, and host ecological covariates.

**Fig. 4:**
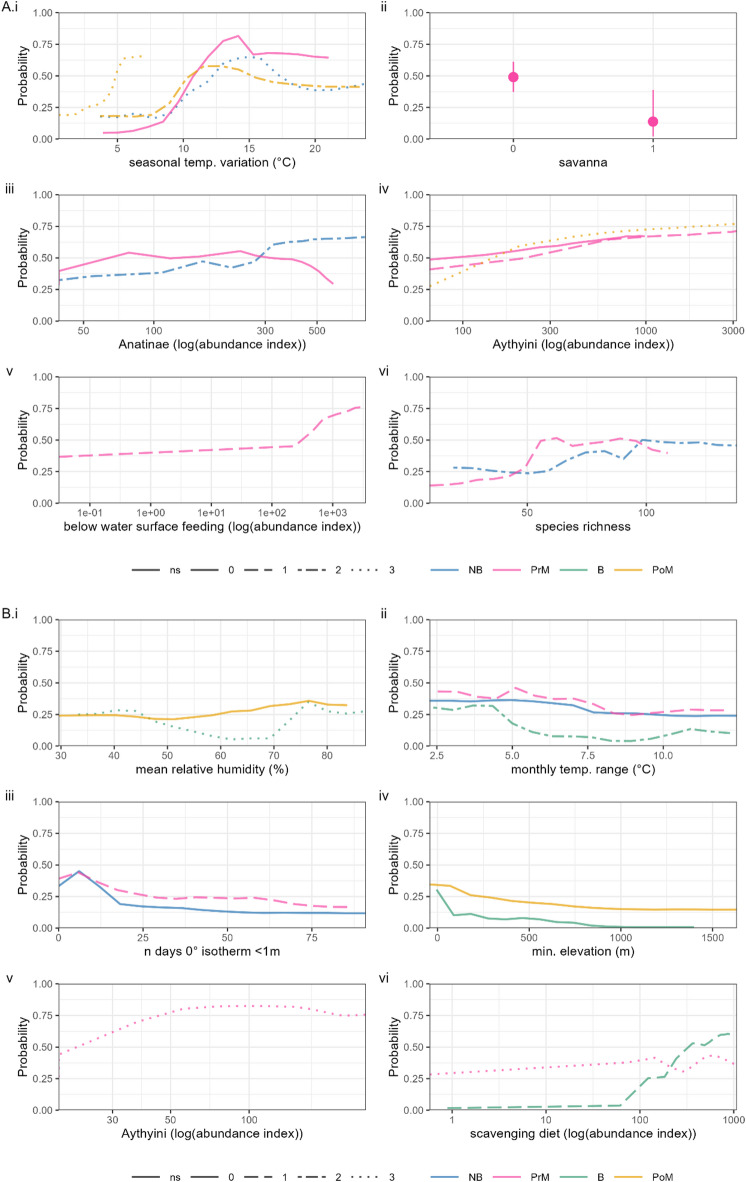
Partial dependence of BART models. Partial dependence associated with top six covariates by average variable importance in final fitted BART models for period (**A**) (10/8/2016 to 9/8/2021, spanning H5N8 and H5N6 events) and period (**B**) (10/8/2021 to 29/2/2024, ongoing H5N1 outbreak). Y axis denotes marginal probability of H5 HPAI presence, i.e., averaging out effects of all other covariates. Solid lines denote median values over 8000 draws from the posterior tree space. Colour denotes separate modelled bird behavioural seasons (NB = Nonbreeding season, PrM = Pre-breeding migration, B = Breeding season, PoM = Post-breeding migration) of H5 HPAI and line type denotes seasonal delay increasing from 0 (solid) to 3 (dotted) seasons prior.

These continental-scale differences can be explained by differing importance of bioclimatic, topographic, livestock demographic, and host ecological factors in dynamics of respective H5 HPAI outbreaks (Figs. 3 and 4). During H5N8 and H5N6 events of HPAI clade 2.3.4.4b, bioclimatic, topographic, and host ecological variables were each well-represented after variable selection in models of each season (Fig. 3A). The presence of lagged variables among the bioclimatic variables retained for period A models pointed to an association between colder months (within nonbreeding season; November to February) and risk. Partial dependence curves showed that more variable temperatures over the respective season led to greater predicted risk of influenza (Fig. 4A.i), and that lower zero-degree isotherms (i.e., freezing temperatures nearer ground level) were associated with risk in nonbreeding and post-breeding migratory seasons (Supplemental Figures S5, S7). No overall topographic patterns dominated, with different variables being retained for different seasons, e.g., savanna (defined as land with 10–30% cover of tree canopies > 2 m) was associated with decreased risk for pre-breeding migration (Fig. 4A.ii) while low-lying, flat land was associated with increased risk for the nonbreeding season (Supplemental Fig. S6).

Each season’s model in period A also emphasised the importance of host ecology, particularly the pre-breeding migration model (Fig. 3A). Many ecological variables retained related to population sizes during the breeding season via lagged effects, suggesting predictable annual cycling in influenza risk. Specifically, abundance indices of Anatinae, Aythyini, and to a lesser extent, Anserinae and Ardeidae showed generally consistent increases in risk with increasing host population (Fig. 4A.iii, iv; Supplemental Figures S5-S7). Behavioural and dietary traits showed minor, season-dependent effects, e.g., risk association with increasing population of underwater feeders (Fig. 4A.v) or predatory birds (Supplemental Fig. S5). Notably, the collective population of migratory species during the breeding season was predictive of risk during the subsequent non-breeding season (Supplemental Figure S5). Risk of H5 HPAI also increased with a more diverse host community in species richness, up to a plateau at ~ 50–100 species (Fig. 4A.vi).

Models trained on geolocated H5N1 HPAI clade 2.3.4.4b data in period B also featured a combination of bioclimatic, topographic, and ecological variables with some key differences. As in period A, winter climate, including humidity, temperature, temperature variation, and zero-degree isotherms of post-breeding and nonbreeding seasons were consistently associated with H5N1 HPAI presence throughout the year (Figs. 3B and 4B.i - iii). For topography, low elevation was again associated with greater risk (Fig. 4B.iv). Compared to period A, however, land cover had additional consistent associations with influenza risk, including greater risk in denser vegetated areas, urban and built-up areas, and coastal areas (Supplemental Figures S8, S9, S11), consistent with topography of the projected risk maps.

Among potential high-risk host taxa, abundance index of Aythyini had the highest average importance in period B models (Fig. 3B), associated with a relatively rapid increase and plateau in predicted influenza risk during the pre-breeding migratory season (Fig. 4B.v). Other taxa had predictive effects with different annual lags e.g., breeding populations of Anatidae and migratory populations of Ardeidae and Anserinae affecting later seasons (Supplemental Figures S8, S9, S11). Dietary and behavioural species-trait abundance indices were retained across all seasons, with partial dependence curves tending to capture sharp risk increases beyond threshold populations, e.g., of scavenging birds (Fig. 3B.vi, Supplemental Figures S8 – S11). A positive risk relationship was again retained with species richness, though only richness during the nonbreeding season was influential (Supplemental Figures S8, S9). Overall, the season-specific effects of these traits suggests that localised ecology (i.e., small-scale interactions between different covariates) is important to understanding the distribution of H5 clade 2.3.4.4b influenza.

**Fig. 5:**
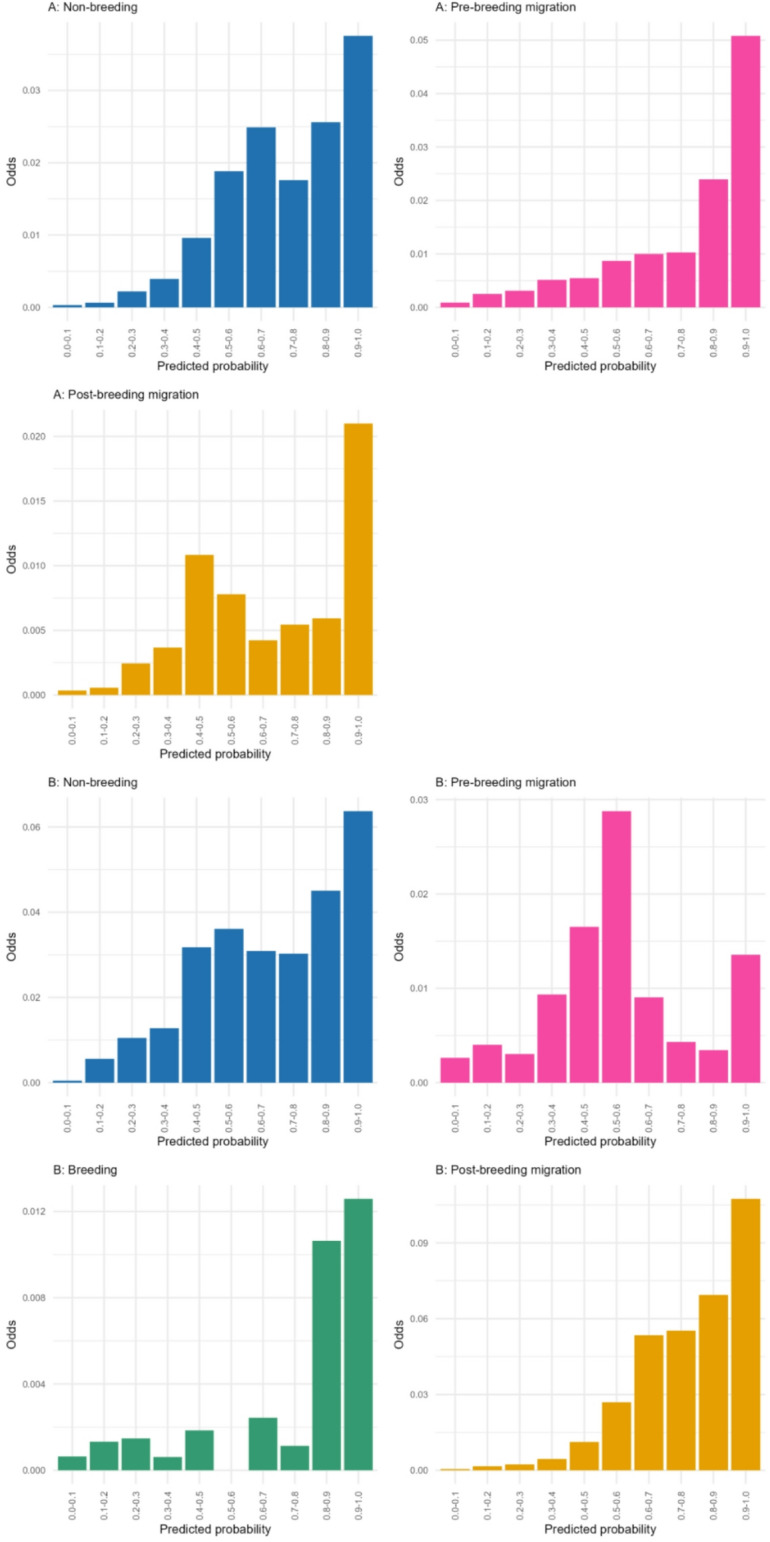
Comparison of odds of reporting H5 clade 2.3.3.4b HPAI in domestic birds stratified by model-predicted risk in wild birds. Odds of H5 infection in domestic birds for each period and season by model predicted probabilities of H5 clade 2.3.4.4b HPAI risk for wild birds.

Our comparison of model predictions with domestic H5 HPAI detections indicated a trend towards increased odds of infection in domestic birds with model-projected risk of H5 HPAI presence in wild birds for both model periods (Fig. 5). Spatial concordance between model-generated predicted probabilities and reports of H5 HPAI in domestic birds is visualised in Supplemental Figures S12 and S13.

## Discussion

In this study we have demonstrated how inclusion of both environmental and ecological covariates can lead to generalisable models of avian influenza distribution in wild birds, particularly in colder months of the year. Our analysis reveals subtle differences in the epidemiology of clade 2.3.4.4b of H5 HPAI once H5N1 became the dominant subtype within Europe. This is reflected both in the projected geospatial risk distributions, and in which covariates best explain these distributions between period A and period B models. Our projections suggest that the ongoing outbreak of the H5N1 genotype is associated with an increased risk of avian influenza presence around northwest Europe, in agreement with the observed large numbers of H5 HPAI detections in this region. Underlying risk patterns were more ambiguous in eastern Europe due to less intensive sampling, though our projections suggest that while H5N8 and H5N6 HPAI widely dispersed across these areas during non-breeding and migratory seasons, the incursion of H5N1 was limited to waterways and shores.

In line with these observed patterns of detection, we expect risk to be associated with proximity to coastlines, low elevations, low temperatures, and presence of high-risk waterbirds. We found that environmental factors reflecting physical geography and climate tended to be more consistent than wild bird ecology in predicting H5 HPAI risk, possibly reflecting inherent constraints on wild bird ecology imposed by background environmental factors. Our results suggest that while risk is indeed highest in the heavily sampled regions of northwest Europe, there is also a strong potential for H5 HPAI presence in regions where case detections are less frequent. From a theoretical perspective, the incorporation of ecological risk factors into H5 HPAI presence/absence models appears to offer a small but distinct improvement in predictive ability.

The minimal differences in performance when adding country-level random intercepts suggest that any spatial heterogeneity in our detection data was effectively captured by weighting for background citizen surveillance of birds in our pseudo-absence generation process. However, the very low numbers of detections in specific eastern European countries (e.g., Albania, Belarus, Moldova, Ukraine) suggest that there are indeed national-level differences in detection and reporting rates which are difficult to estimate due to the absence of information from these countries in many of our training datasets. The selection of many cross-seasonal covariates during model fitting suggests that future mechanistic models may need to incorporate delay effects to fully capture the complex dynamics of avian influenza outbreaks in wild birds.

Our risk projections point to an increased risk of H5 HPAI presence in northwest Europe, particularly around the North Sea coast, associated with the emergence of the H5N1 genotype of clade 2.3.4.4b. The confidence limits for our projections suggest that the Black Sea coast may also be a zone of high H5N1 risk, as it was for the previous H5N8 subtype of clade 2.3.4.4b. This shift in geographical burden of risk appears to be driven by differing importance of climate, geography, and abundance of specific bird taxa (Anatinae, Anserinae, Ardeidae, and Aythyini) at different calendar times and with different seasonal lags. The narrower model uncertainty and greater predictability seen for colder seasons, especially in period B models of H5N1 (Table 4), might imply more consistent and less stochastic transmission dynamics occurring as multiple bird species co-congregate during wintering. However, we note these seasons had substantially more training data available for period B.

SDMs trained on clades prior to 2.3.4.4b have established a consistent link between temperature and avian influenza in wild birds^48,49,51,52^, with further study highlighting the association between both temperature-driven waterbird movements and congregation of waterbirds at the zero-degree isotherm and the distribution and spread of HPAI^60^. We report this association with temperature and zero-degree isotherm variables also holds for H5 clade 2.3.4.4b in our models. At least one temperature variable was selected for all models and temperature variation had high variable importance across both periods A and B, whilst zero-degree isotherm variables were selected for all bird behavioural seasons except pre-breeding migration in period A and the breeding season in period B. As for other environmental variables in our analysis, these climatic covariates are likely to have multifactorial influences on H5 HPAI risk in wild birds, either directly, e.g., upon environmental stability of virus particles, or through indirect hierarchical causality, e.g., upon habitat suitability for high-risk hosts or bird population mixing as water sources thaw post-winter^30,31,60^. While we attempt to capture several mechanistic routes by explicitly including host taxonomic and behavioural traits, disentangling the contributions of each to transmission needs localised evidence. We assert that macroecological models such as ours can highlight promising avenues for mechanistic study, e.g., of how isotherm shifts change high-risk water-feeding behaviours (Figs. 3 and 4), and we would advocate for more such observational or experimental field studies – particularly in wild bird population centres we project as most prone to HPAI (Fig. 2).

The importance of climatic variables in our models also suggests that anthropogenic climate change is likely to drive changes in the spatiotemporal distribution of avian influenza in Europe. Relatedly, we find minor, but detectable effects of land cover type on HPAI risk, with lower average risk in savanna-type or mixed-forest biomes and greater average risk in urban or built-up lands. This may hint at a role of anthropogenic habitat disruption and fragmentation upon influenza epidemiology, as empirically shown for many other zoonotic disease systems^87^.

By conducting variable selection we demonstrate that incorporating ecology provides a unique improvement in models of avian influenza presence; all models featured ecological covariates, with most combining a range of different bird population indices. This identifies wild bird ecology as a distinct source of information not present in background environmental data. In contrast to the more consistent set of climate covariates, ecological covariates varied seasonally with exact indicator taxa and/or trait abundance indices preferentially being retained in models of different bird behaviours and with different temporal lags. This suggests that avian influenza incidence is primarily driven by patterns of host species-level climatic and environmental suitability but is also subject to highly localised ecological factors for H5 clade 2.3.4.4b.

We observed an association with bird species richness for nonbreeding and pre-breeding migratory seasons. Whether host diversity generally increases or decreases disease risk is somewhat contested^88^ and depends on scale, pathogen traits, and host species composition. We report a positive relationship between species richness and H5 HPAI presence, potentially capturing greater opportunities for contact and transmission between different populations^28^ and/or importance of additional key host taxa following Huang et al. reporting a similar positive effect of richness of “higher-risk hosts” upon H5 HPAI in Europe from 2005 to 2008^50^.

Among host taxa we explicitly modelled, relative abundance of Anatinae (dabbling ducks) was the most consistent predictor of H5 HPAI presence, followed by Aythini (pochards), Anserinae (swans and geese), and Ardeidae (herons). Except for Ardeidae, these taxa are generally considered maintenance hosts of avian influenza as part of the Anseriformes. Aside from topographic variables like elevation and distance to wetland, the most informative predictors of avian influenza in SDMs for Japan and South Korea were population measures or correlates of Anatidae^53,54^. Mallards within the Anatidae appear to be effective carriers as they may experience only mild disease, even when infected with H5N1 clade 2.3.4.4b^25^. In phylogenetic discrete trait analyses of the North American H5N1 clade 2.3.4.4b outbreak, longer persistence of infection and high rates of transmission to other taxa were observed for Anseriformes, as well as a Charadriiformes^22^. Despite this, model variable selection did not retain any abundance indices of Charadriiformes hosts (Laridae (gulls), *Arenaria* (turnstones), or *Calidris* (sandpipers)). This suggests that while gulls and shorebirds have experienced unprecedented infection and die-offs, their changing seasonal abundances do not appear to inform H5 HPAI presence beyond environmental conditions. Recent phylodynamic analyses have confirmed the epidemiological importance of Charadriiformes in reassortment emergence and intercontinental spread of H5 HPAI clade 2.3.4.4b^89^. Although the total abundance index of migratory birds was only retained in a single model of period A, the effects of migration may be reflected in the changing predictive power of these taxa throughout the calendar year.

Alongside the abundance estimates for high-risk taxa, our construction of species-trait abundance indices represents a novel attempt to quantify the effect of ecological factors on H5 HPAI risk at the continental scale. Although they improved models, overall abundance indices of birds with specific diets and foraging behaviours showed varying and highly localised trends in risk (Supplemental Figures S5 – S11). Foraging behaviour has previously been suggested as a major driver of avian influenza^28^, as faecal-oral transmission primarily occurs through viral shedding into water. However, studies looking at viral shedding of HPAI including clade 2.3.4.4b viruses have reported high levels of additional shedding via the respiratory system^32–35^. We find a somewhat unexpected importance of scavenging and predation behaviour in both modelled periods. Combined with the high reported rates of cross-species transmission to predatory birds such as owls and raptors^22^, this indicates the role of predation in spatial transmission should not be ignored.

Equivalent functional ecology and life history measures have been found to act as predictors of individual-level infection with previous clades^50,90^, and emerging studies are highlighting similar trends for community-level infection, where plant consumption was predictive of H5 HPAI clade 2.3.4.4b infection^27^, consistent with our findings for H5N1 in period B. The relatively low importance assigned to functional ecological factors in our models is potentially due to a degree of redundancy in the models with both taxon abundance layers and species-trait abundance layers; the derived nature of the species-trait abundance layers means that they carry an inherently higher level of error than the taxon abundance layers, while contributing a limited amount of independent information compared to the taxon abundance layers. For instance, knowing the spatial abundance of specific high-risk waterfowl taxa may contribute the same information as knowing the combined species-trait abundance layers for plant-feeding, feeding around the water surface, and feeding below the water surface.

Continental-scale modelling efforts to understand avian infectious diseases are critically necessary, given their ability to disseminate long distances through bird migration^26^; recent phylodynamic models estimate it took only five months for a single introduction of clade 2.3.4.4b H5 HPAI to North America to spread from east coast to west coast via migratory flyways^22^. However, if we are to better capture host dynamics at these scales and improve spatial projections of avian influenza, we need higher-quality underlying ecological data. The spatial ecology layers we use come with several important caveats. In the absence of comprehensive survey data, we use projected abundance layers from eBird Status and Trends products and global species population size estimates. Our models are therefore effectively downstream from another set of models and thus need to be considered in light of any uncertainties and limitations associated with those models^91^. The projections from eBird Status and Trends also only covered a subset of all bird species known to be present in Europe, meaning that our ecological covariates could potentially underestimate abundances in spatially heterogeneous ways. The role of ecological covariates in H5 HPAI risk prediction points to high-confidence models of wild bird abundance for a more comprehensive selection of species and covering a wider geographical area as a key target for future research. Automated “computer vision” systems to recognise presence, abundance, and location of species from digital images and their metadata could prove useful in this context^92^.

There are also substantial challenges associated with working with current standards of HPAI case reports. Although HPAI is classified as a listed disease by the WOAH meaning that national authorities are required to report HPAI viruses detected in both poultry and non-poultry species, including wild birds^93^, surveillance strategies across countries are not standardised and may differ substantially between countries in the same region^94,95^. This results in significant underreporting of wild bird HPAI, with gaps increasingly evident in the wake of the expansive wild bird die-off associated with clade 2.3.4.4b^96^. Passive surveillance is frequently used and relies on submission of sick or dead wild birds. Rates of data submission are likely to show spatial and temporal variation based on factors such as human population density and public awareness of disease, whilst testing capacity may also vary over time depending on factors such as disease priority and demand. Similar variation also exists across active surveillance strategies. In this study we responded to these data quality issues by considering presence/pseudo-absence at the geographic level rather than finer-grained reported incidence data, which are associated with inherently higher levels of error due to the more complex nature of the signal being measured. Consistency in surveillance both within and between countries, reporting of negative tests and/or submission rates from passive surveillance, and accurate and consistent labelling of affected species would significantly improve data interpretability for end users. Higher quality of reporting data should improve the predictive ability of complex mechanistic models, as well as broader presence/absence approaches as in our work.

Our findings also have translational value for One Health protection. While correlative, our continental mapped projections of H5 HPAI risk are easily interpretable. A key benefit of choosing a Bayesian framework is its intuitive treatment of uncertainty, which can itself be mapped to distinguish sites that are not only high-risk but predicted so with most certainty (Fig. 2). These sites can shape targeted policy, most obviously in directing wild bird surveillance, particularly in those areas predicted to be H5 HPAI hotspots despite having very little reported sampling such as the eastern Balkans (Figs. 2 and 3). Equally, these predicted hotspots could also inform likely sites for surveillance of mammalian spillovers, which have been observed in Europe among wild carnivores^97^ and more recently, livestock^98^. Beyond surveillance, our models may be used in conjunction with more traditional epidemiological time series to strategically determine which regions’ poultry holders or gamekeepers should be primed with advisory notices or information to prepare ahead of potential housing orders or other control measures in response to first alerts of active transmission. This could also extend towards targeting livestock holders for health protection considering the known (although infrequent) human infectivity of H5 HPAI clade 2.3.4.4b viruses and their evolutionary lability. However, we note that human exposure remains primarily occupational and any spatial patterns of zoonotic cases are likely to be obscured by poultry and other livestock movement networks.

In this study we have identified a shift in wild bird H5 HPAI risk towards northwest Europe following the dominance of the H5N1 genotype of clade 2.3.4.4b, which is associated with changes to the underlying predictive factors driving H5 HPAI epidemiology. Our work also serves as an assessment of the role of ecological factors in predictive models of HPAI, with our results suggesting that these factors can improve model performance, particularly if more accurate quantitative measures of wild bird ecology become available in the future.

## Supplementary Information

Below is the link to the electronic supplementary material.

Supplementary Material 1

## Data Availability

All raw data on avian influenza records and environmental and ecological predictor variables are available from secondary sources cited within the manuscript. A separate data repository is available for our processed data records at https://zenodo.org/records/15344899. All code to reproduce data collection, processing and analysis is available at https://github.com/sarahhayes/avian_flu_sdm.
