# Supplementary material for: Ecology and environment predict spatially stratified risk of H5 highly pathogenic avian influenza clade 2.3.4.4b in wild birds across Europe
Source: Sci Rep. 2025 Dec 2;16:997. doi: 10.1038/s41598-025-30651-9 (PMC12783809; doi:10.1038/s41598-025-30651-9)
Supplement: Supplementary file 1 — Supplementary Material 1 [file 41598_2025_30651_MOESM1_ESM.docx]

**Supplementary information for Hayes, Hilton et al.: ‘Ecology and environment predict spatially stratified risk of H5 highly pathogenic avian influenza clade 2.3.4.4b in wild birds across Europe’**

**
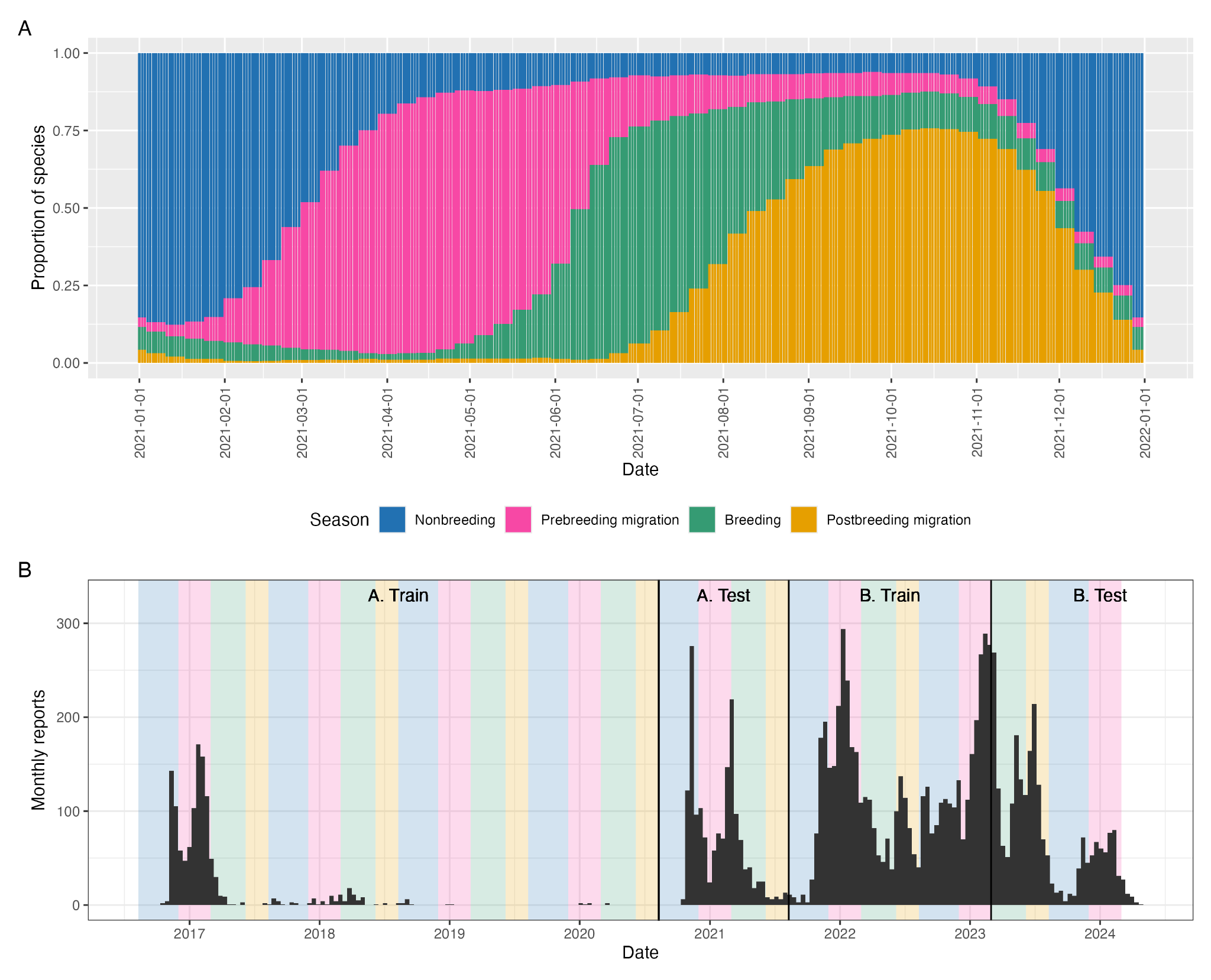
**

**Supplemental Figure S1. Aggregated Europe-wide bird behavioural seasons.**

A) Distribution of behavioural season of seasonal European bird species over the course of the calendar year 2021, based on eBird Status and Trends records. B) Total independent incident reports of H5 HPAI clade 2.3.4.4b in wild birds during model training/test periods, with shaded time strips denoting annual seasonal periods as chosen considering panel A, demonstrating the well-established persistence during warmer breeding and post-breeding migration seasons associated with H5N1 HPAI during period B.


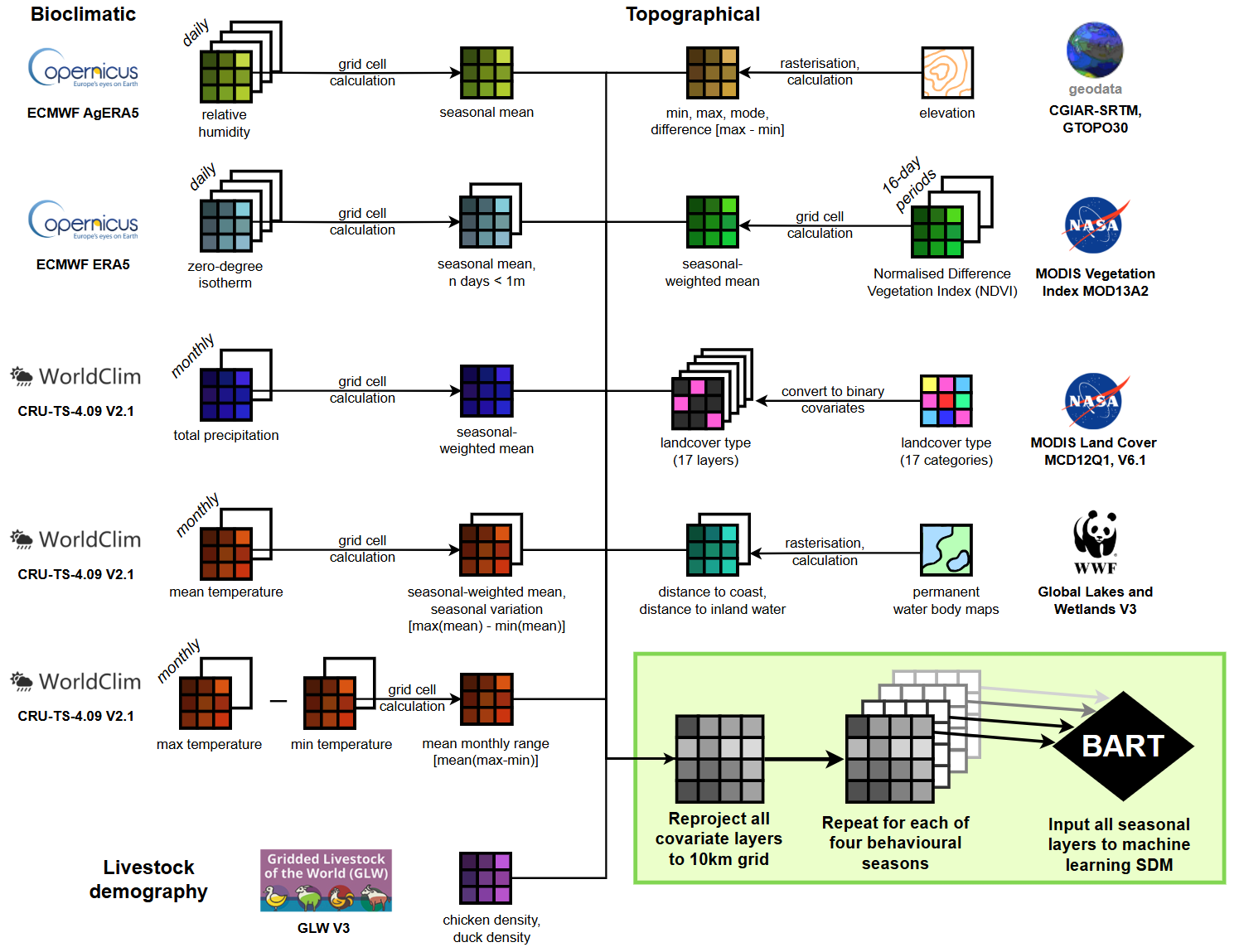


**Supplemental Figure S2. Data processing schematic for environmental covariates.**

Data processing schematic depicting source, type, and temporal availability of each raw environmental data layer, before processing, recalculation, and reprojection into final layers as model-ready inputs. Shaded grids denote individual raster data layers. BART = Bayesian Additive Regression Trees, CGIAR = (formerly) Consultative Group for International Agricultural Research, CRU-TS = Climatic Research Unit Time Series, ECMWF = European Centre for Medium-Range Weather Forecasts, MODIS = Moderate Resolution Imaging Spectroradiometer, SDM = Species Distribution Model, STRM = Shuttle Radar Topography Mission, WWF = World Wildlife Fund.

**
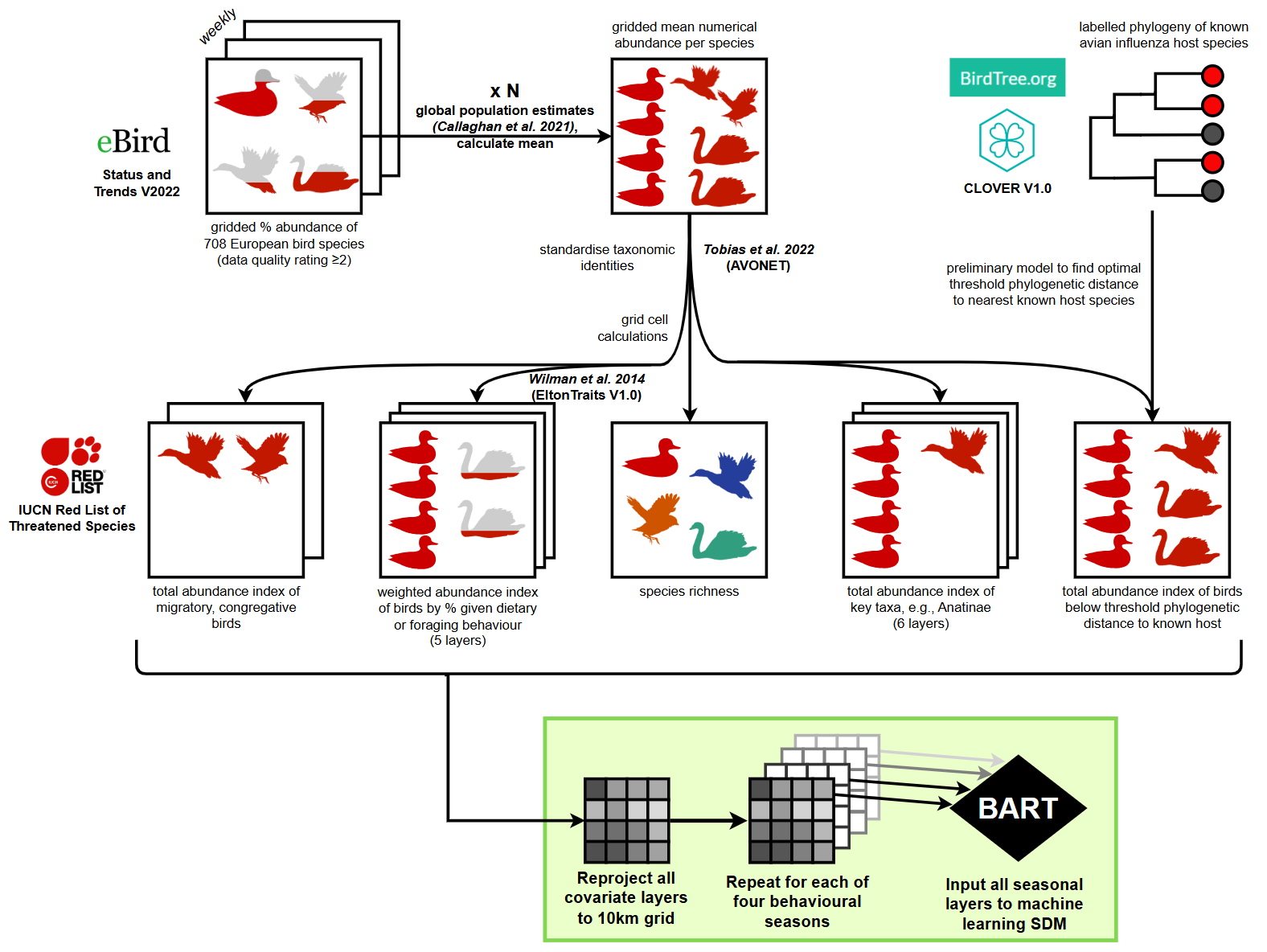
**

**Supplemental Figure S3. Data processing schematic for wild bird ecological covariates.**

Data processing schematic depicting source, type, and temporal availability of raw wild bird ecological data. Percentage abundance measures are first converted to numerical abundance measures, before generating multiple raster data layers that aggregate proxy total abundance of individuals exhibiting particular traits by cross-referencing external datasets. BART = Bayesian Additive Regression Trees, IUCN = International Union for Conservation of Nature, SDM = Species Distribution Model.

**
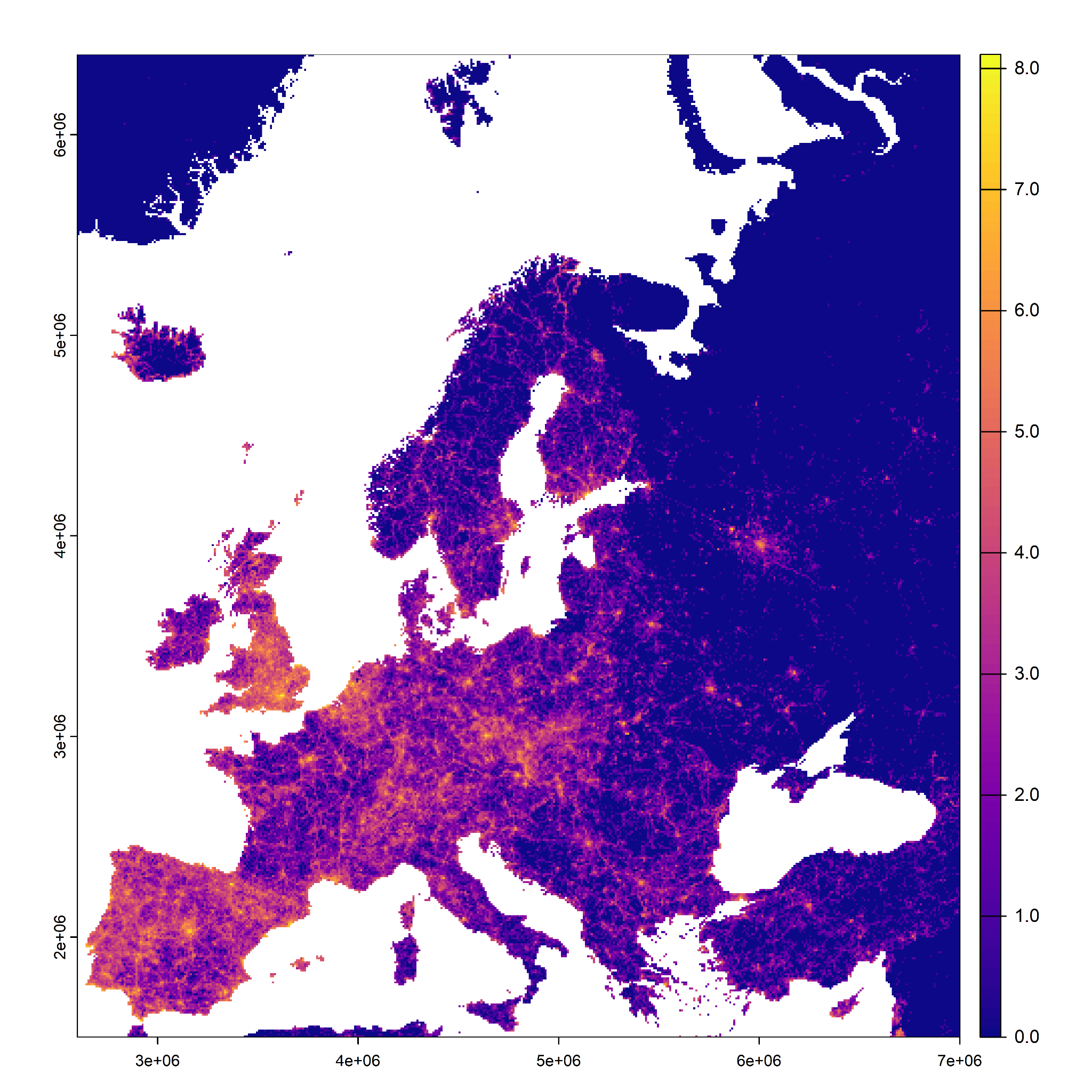
**

**Supplemental Figure S4. Mapped density of eBird sightings.**
Mapped density of bird sighting records in Europe from the eBird citizen surveillance platform. Log-transformed total sightings were counted as instances of unique data-user-geolocations over duration of study period (10/8/2016 - 29/2/2024).

**
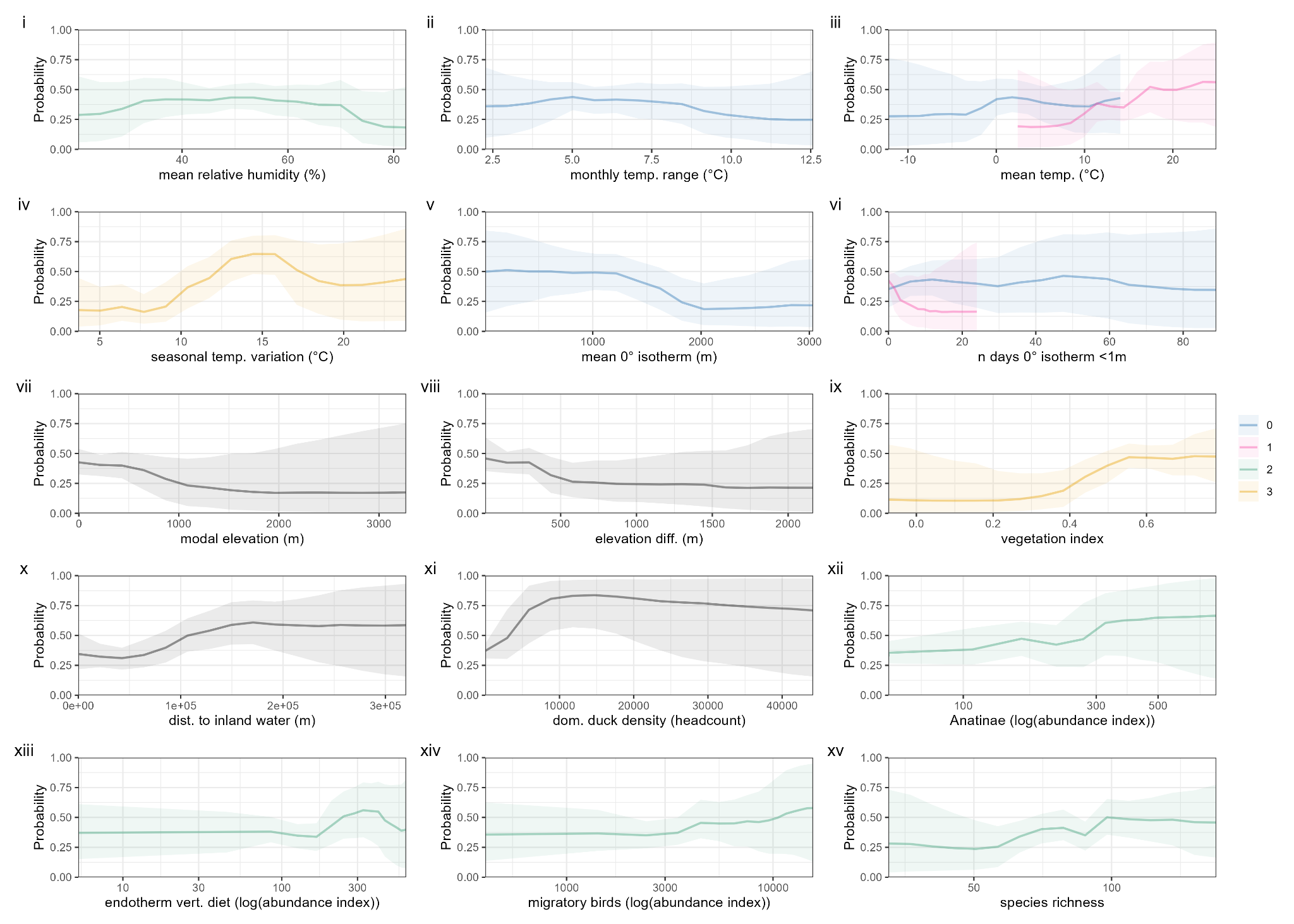
**

**Supplemental Figure S5. Full partial dependence of BART model trained on dataset A, non-breeding season.**
Partial dependence associated with all covariates in final BART model of aggregated non-breeding season (30th November - 28th February) fitted to geospatial H5 HPAI data for period A (10/8/2016 to 9/8/2021, spanning H5N8 and H5N6 events). Y axis denotes marginal probability of H5HPAI presence, i.e., averaging out effects of all other covariates. For continuous covariates, solid lines denote median values while shaded areas denote uncertainty via 2.5th percentile and 97.5th percentile values over 8000 draws from the posterior tree space. For categorical covariates, points denote median values while error bars denote via 2.5th percentile and 97.5th percentile values. Colours denote seasonal delay increasing from 0 (predicted season) to 3 (three seasons prior to predicted season); black denotes non-seasonally-variable covariates.


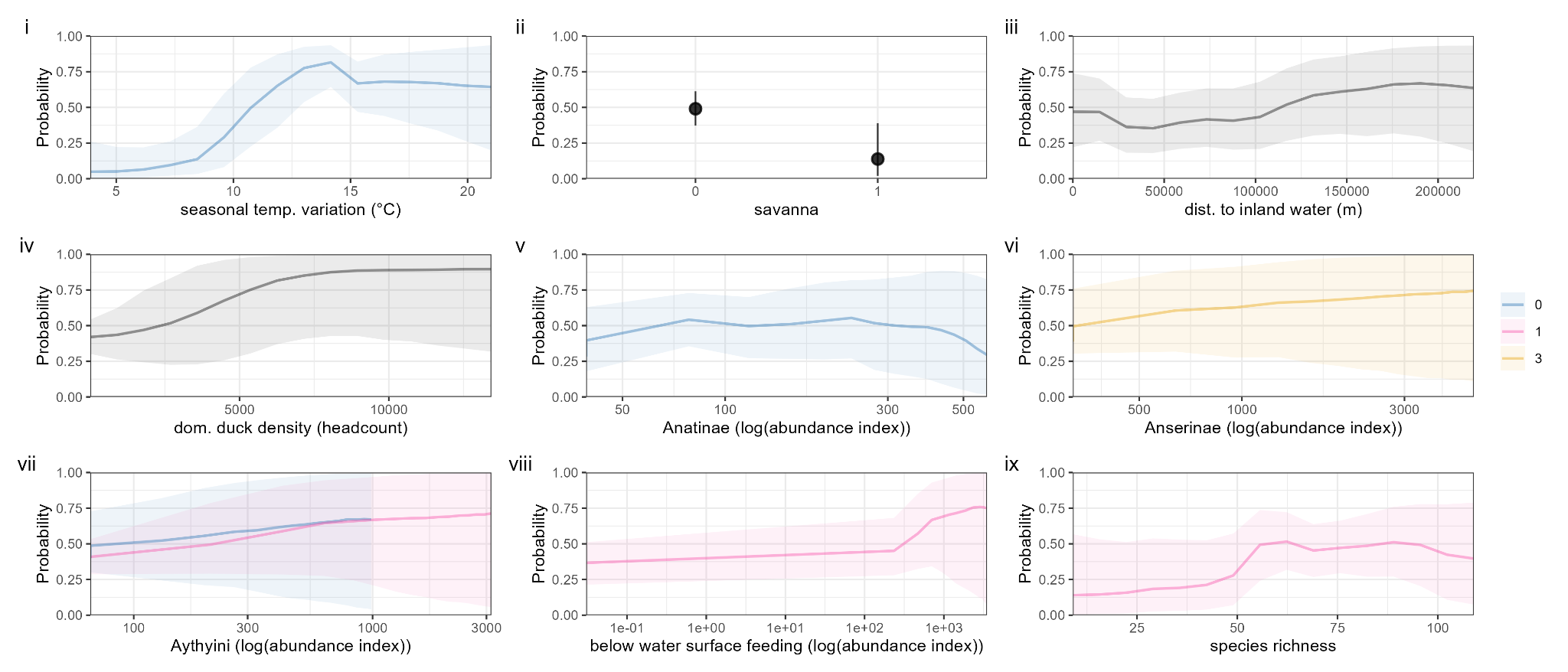
**Supplemental Figure S6. Full partial dependence of BART model trained on dataset A, pre-breeding migration.**
Partial dependence associated with all covariates in final BART model of aggregated pre-breeding migration season (1st March - 6th June) fitted to geospatial H5 HPAI data for period A (10/8/2016 to 9/8/2021, spanning H5N8 and H5N6 events). Y axis denotes marginal probability of H5 HPAI presence, i.e., averaging out effects of all other covariates. For continuous covariates, solid lines denote median values while shaded areas denote uncertainty via 2.5th percentile and 97.5th percentile values over 8000 draws from the posterior tree space. For categorical covariates, points denote median values while error bars denote via 2.5th percentile and 97.5th percentile values. Colours denote seasonal delay increasing from 0 (predicted season) to 3 (three seasons prior to predicted season); black denotes non-seasonally-variable covariates.

**
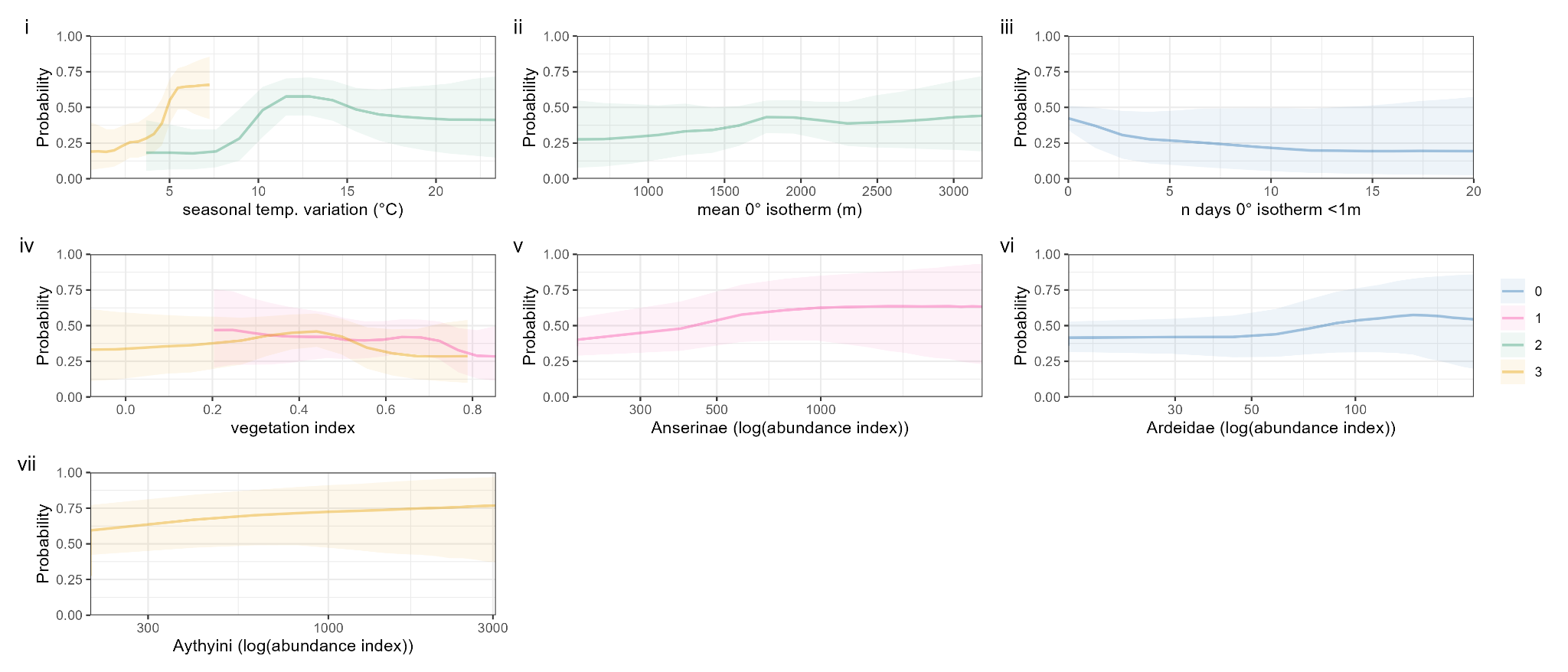
Supplemental Figure S7. Full partial dependence of BART model trained on dataset A, post-breeding migration.**
Partial dependence associated with all covariates in final BART model of aggregated post-breeding migration season (10th August - 29th November) fitted to geospatial H5 HPAI data for period A (10/8/2016 to 9/8/2021, spanning H5N8 and H5N6 events). Y axis denotes marginal probability of H5 HPAI presence, i.e., averaging out effects of all other covariates. For continuous covariates, solid lines denote median values while shaded areas denote uncertainty via 2.5th percentile and 97.5th percentile values over 8000 draws from the posterior tree space. For categorical covariates, points denote median values while error bars denote via 2.5th percentile and 97.5th percentile values. Colours denote seasonal delay increasing from 0 (predicted season) to 3 (three seasons prior to predicted season); black denotes non-seasonally-variable covariates.

**
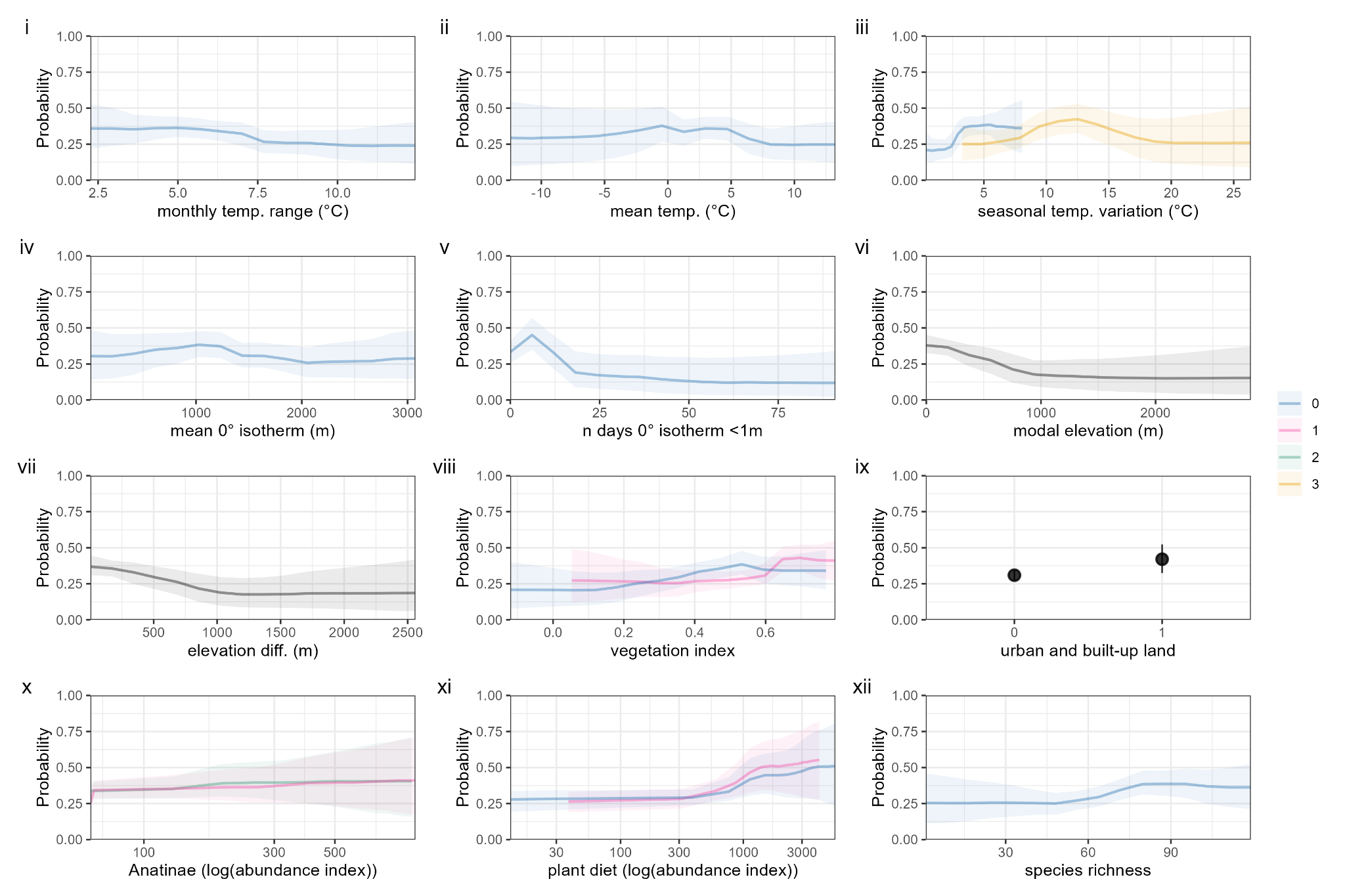
Supplemental Figure S8. Full partial dependence of BART model trained on dataset B, non-breeding season.**
Partial dependence associated with all covariates in final BART model of aggregated non-breeding season (30th November - 28th February) fitted to geospatial H5 HPAI data for period B (10/8/2021 to 29/2/2024, ongoing H5N1 outbreak). Y axis denotes marginal probability of H5 HPAI presence, i.e., averaging out effects of all other covariates. For continuous covariates, solid lines denote median values while shaded areas denote uncertainty via 2.5th percentile and 97.5th percentile values over 8000 draws from the posterior tree space. For categorical covariates, points denote median values while error bars denote via 2.5th percentile and 97.5th percentile values. Colours denote seasonal delay increasing from 0 (predicted season) to 3 (three seasons prior to predicted season); black denotes non-seasonally-variable covariates.

**
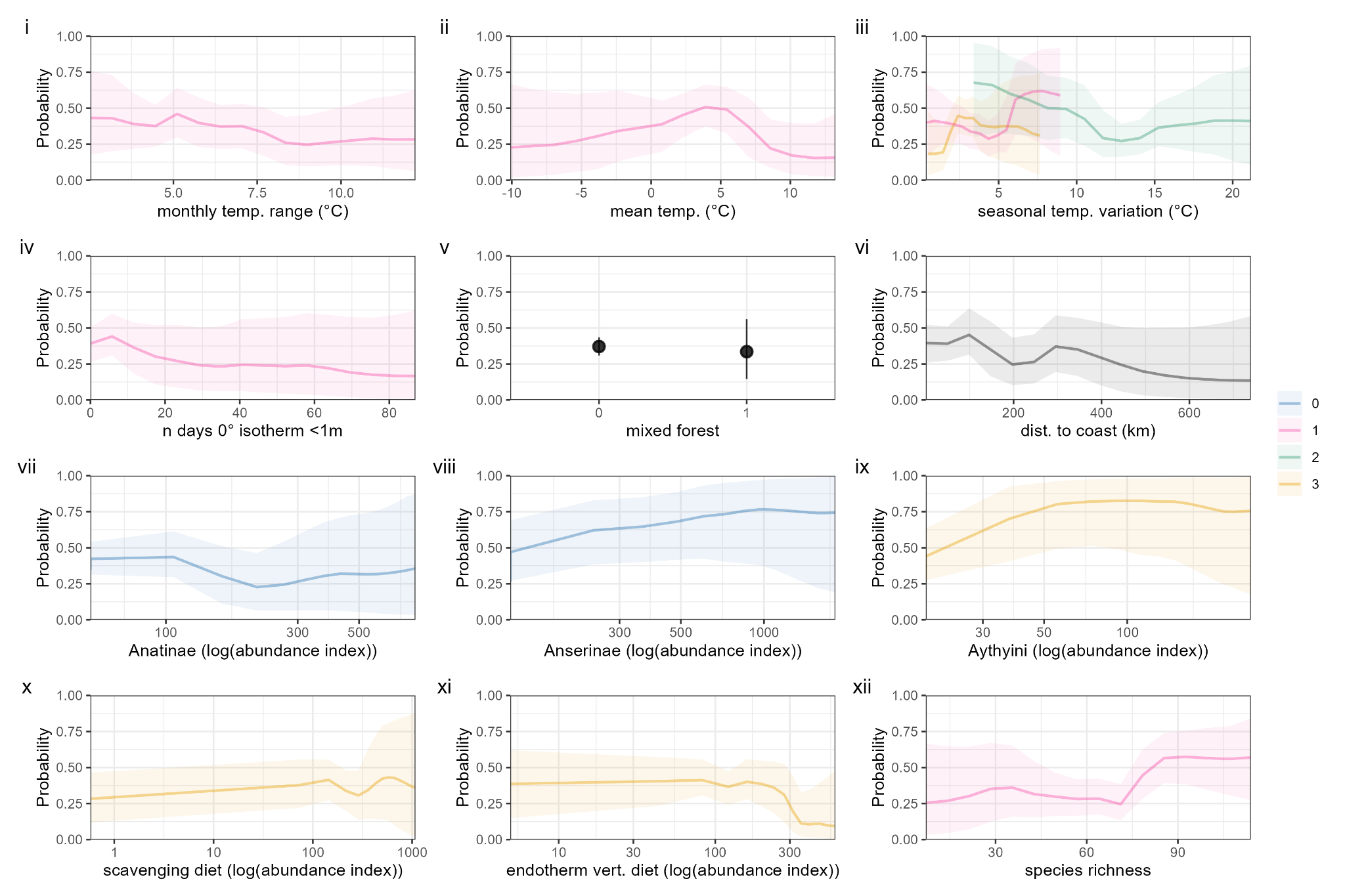
Supplemental Figure S9. Full partial dependence of BART model trained on dataset B, pre-breeding migration.**
Partial dependence associated with all covariates in final BART model of aggregated pre-breeding migration season (1st March - 6th June) fitted to geospatial H5 HPAI data for period B (10/8/2021 to 29/2/2024, ongoing H5N1 outbreak). Y axis denotes marginal probability of H5 HPAI presence, i.e., averaging out effects of all other covariates. For continuous covariates, solid lines denote median values while shaded areas denote uncertainty via 2.5th percentile and 97.5th percentile values over 8000 draws from the posterior tree space. For categorical covariates, points denote median values while error bars denote via 2.5th percentile and 97.5th percentile values. Colours denote seasonal delay increasing from 0 (predicted season) to 3 (three seasons prior to predicted season); black denotes non-seasonally-variable covariates.

**
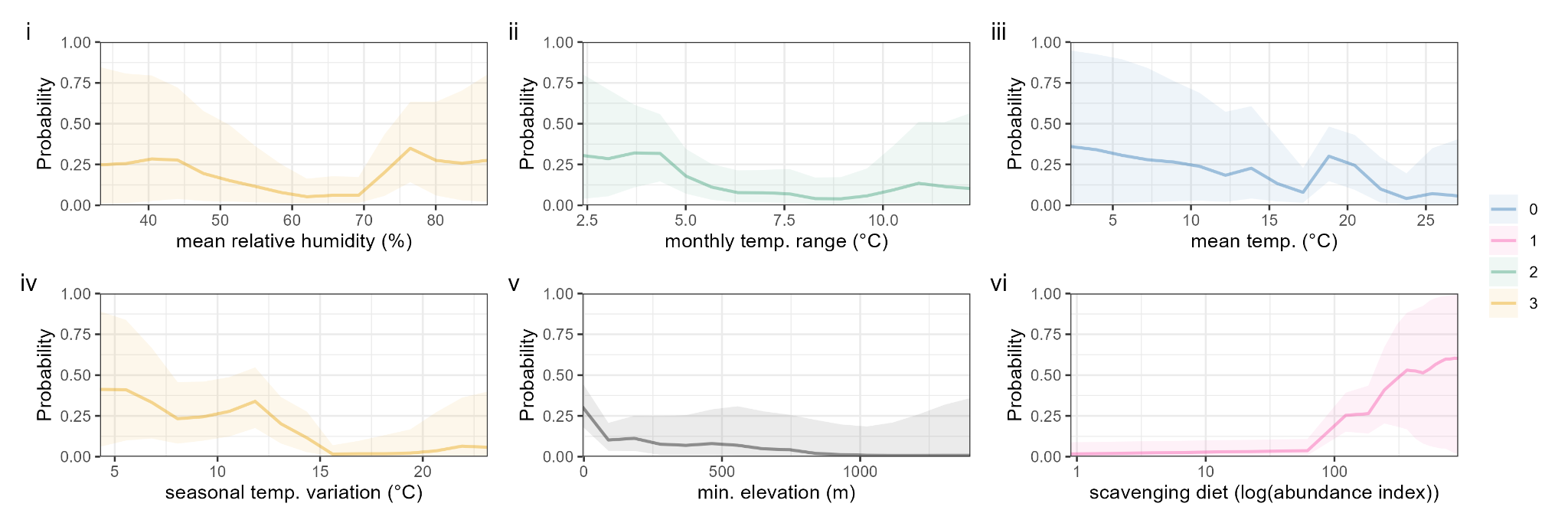
Supplemental Figure S10. Full partial dependence of BART model trained on dataset B, breeding season.**
Partial dependence associated with all covariates in final BART model of aggregated breeding season (7th June - 9th August) fitted to geospatial H5 HPAI data for period B (10/8/2021 to 29/2/2024, ongoing H5N1 outbreak). Y axis denotes marginal probability of H5 HPAI presence, i.e., averaging out effects of all other covariates. For continuous covariates, solid lines denote median values while shaded areas denote uncertainty via 2.5th percentile and 97.5th percentile values over 8000 draws from the posterior tree space. For categorical covariates, points denote median values while error bars denote via 2.5th percentile and 97.5th percentile values. Colours denote seasonal delay increasing from 0 (predicted season) to 3 (three seasons prior to predicted season); black denotes non-seasonally-variable covariates.

**
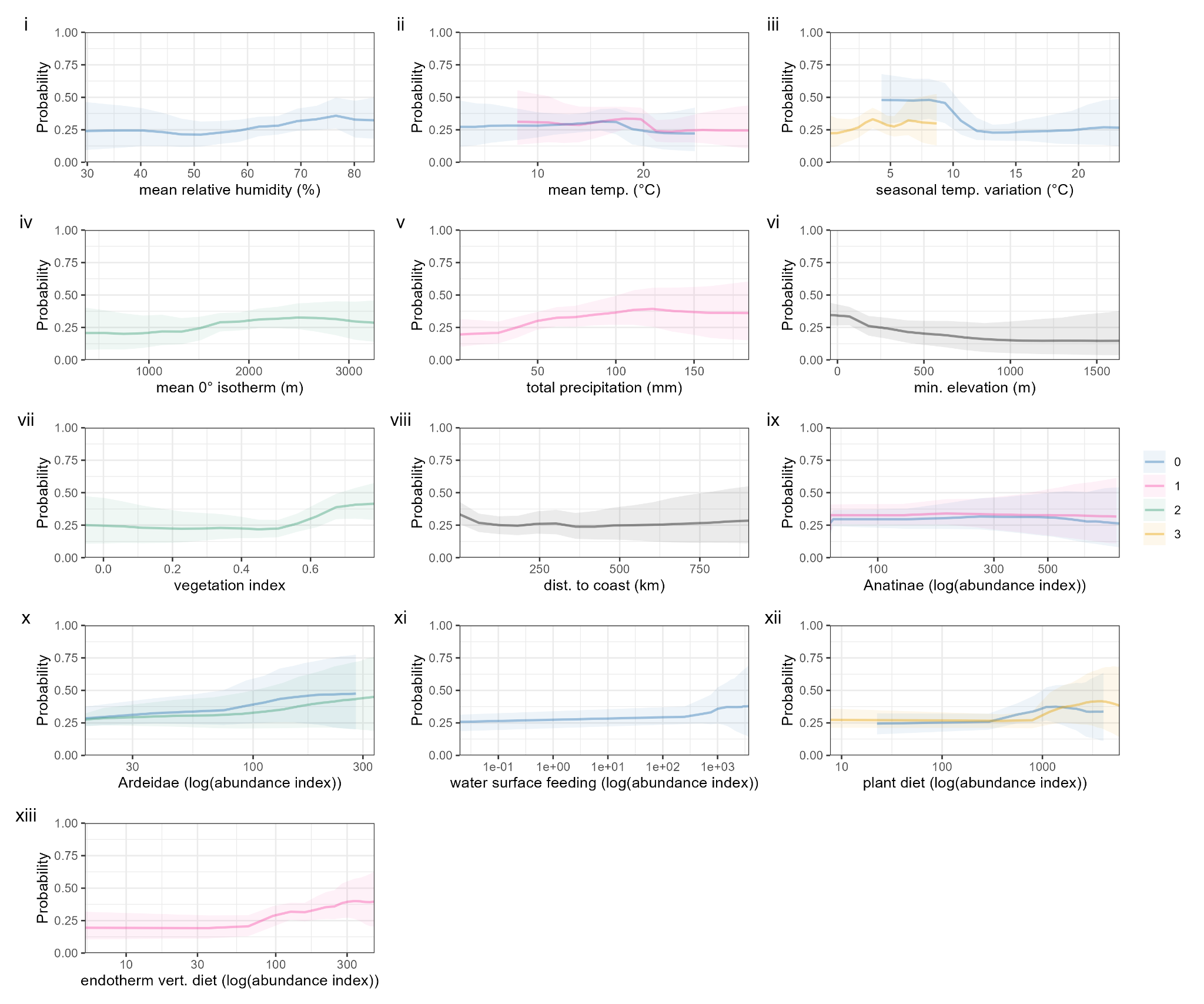
Supplemental Figure S11. Full partial dependence of BART model trained on dataset B, post-breeding migration.**
Partial dependence associated with all covariates in final BART model of aggregated post-breeding migration season (10th August - 29th November) fitted to geospatial H5 HPAI data for period B (10/8/2021 to 29/2/2024, ongoing H5N1 outbreak). Y axis denotes marginal probability of H5 HPAI presence, i.e., averaging out effects of all other covariates. For continuous covariates, solid lines denote median values while shaded areas denote uncertainty via 2.5th percentile and 97.5th percentile values over 8000 draws from the posterior tree space. For categorical covariates, points denote median values while error bars denote via 2.5th percentile and 97.5th percentile values. Colours denote seasonal delay increasing from 0 (predicted season) to 3 (three seasons prior to predicted season); black denotes non-seasonally-variable covariates.


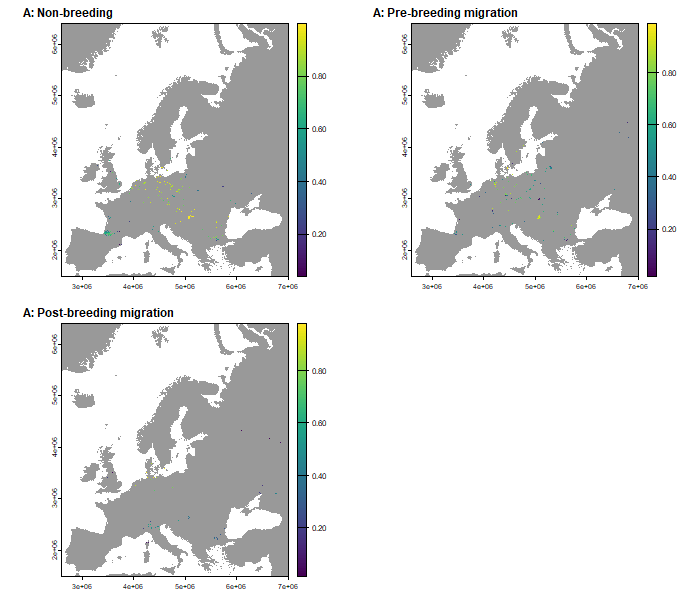


**Supplemental Figure S12. Domestic bird cases and model-predicted wild bird risk, period A.**Maps of the study area with cells containing H5 HPAI in domestic birds highlighted and coloured by the predicted probability from BART models over period A (10/8/2016 to 9/8/2021, spanning H5N8 and H5N6 events). Panels denote individual seasons.


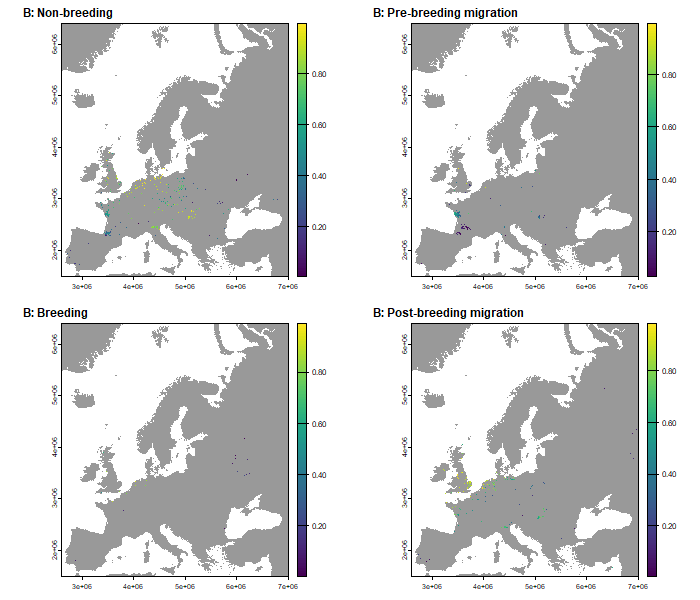


**Supplemental Figure S13. Domestic bird cases and model-predicted wild bird risk, period B.**

Maps of the study area with cells containing H5 HPAI in domestic birds highlighted and coloured by the predicted probability from BART models over period B (10/8/2021 to 29/2/2024, ongoing H5N1 outbreak). Panels denote individual seasons.

**Supplemental Methods S1.**

**Rationalising taxonomic naming**

Taxonomic labelling was not consistent between our species-level data sources, meaning that certain species identified by eBird as being present in Europe did not appear in some of the other data sources, despite these sources theoretically being more comprehensive than the eBird Status and Trends data (which is limited to those species which are confidently modelled by eBird). To remedy this we performed a semi-automated rationalisation process using the AVONET database of bird species names[^1^](https://www.zotero.org/google-docs/?nGDjJ7). Each datapoint in the AVONET database corresponds to a single species and includes binomial names from three different resources (BirdLife, eBird, and BirdTree) as well as an alphanumeric ID string, allowing for identification of synonyms when comparing data from sources using differing taxonomies. For each species identified by eBird as being present in Europe (obtained by filtering by region within the eBird status and trends data) we used AVONET to generate a set of possible synonyms for that species. We matched eBird species abundance records of a species with species-level trait data from other datasets by first searching for that species’ binomial name in that dataset, and then searching for its synonyms if the binomial name was missing. Where the synonyms were absent or where multiple synonyms for a species appeared in the trait data, we checked the data manually and used a combination of renaming and removal of problematic species to ensure that each species had exactly one match in each trait dataset, based on the Avibase online bird taxonomy database[^2^](https://www.zotero.org/google-docs/?Jkmcbv).

Our species-level trait data construction begins by loading in 708 species records from eBird Status and Trends. We then bring in the list of global species population size estimates from Callaghan *et al.*[^3^](https://www.zotero.org/google-docs/?Fmta6u), and for each species in the eBird Status and Trends data attempt to assign a population size. The following 6 species in the eBird list could not be matched to any records in the population size estimate data:

- Northern Hawk Owl, *Surnia ulula*
- Iberian Green Woodpecker, *Picus sharpei*
- Western Subalpine Warbler, *Curruca iberiae*
- Kruper's Nuthatch, *Sitta krueperi*
- Amur Stonechat, *Saxicola stejnegeri*
- Eastern Black-eared Wheatear, *Oenanthe melanoleuca*

We removed *Surnia ulula*, *Sitta krueperi*, and *Saxicola stejnegeri* from our species list as we were unable to find possible matches in the population size estimate data. *Picus sharpei* and *Oenanthe melanoleuca* are classified in some sources as subspecies of other species found in eBird (*Picus viridis*, the European green woodpecker, and *Oenanthe hispanica*, the Western black-eared wheatear, respectively). In these cases it was necessary to remove both species in each pair from our species list because the total *Picus viridis* population size in Callaghan *et al.* effectively included both *Picus viridis* and *Picus hispanica*, meaning that keeping *Picus viridis* in the model would result in us effectively overestimating the numbers of *Picus viridis* present outside of *Picus hispanica*’s range. The same considerations motivated us to remove both *Oenanthe melanoleuca* and *Oenanthe hispanica*. The genus *Curruca* has only recently been recognised and does not appear in some sources and so we remove all of its members. After these removals we were left with 691 of the initial 708 species. Checking the CLOVER database with scientific names and pseudonyms from AVIBASE revealed that none of the species we removed were known avian influenza hosts.

After matching the species from eBird Status and Trends to population size estimates, we used the AVONET database to identify possible species synonyms. The following species could not be found in AVONET:

- Grey-headed Swamphen, *Porphyrio poliocephalus*
- Yellow-headed caracara, *Daptrius chimachima*
- Oriental cuckoo, *Cuculus optatus*

Consulting eBird suggested that *Porphyrio poliocephalus* is found only in small numbers on the outskirts of Europe[^4^](https://www.zotero.org/google-docs/?Wf9X4n), and so we removed it from our species list. We replaced *Daptrius chimachima* with *Milvago chimachima*, which is synonymous with *Daptrius chimachima*[^5^](https://www.zotero.org/google-docs/?8VDW19). Although *Cuculus optatus* was absent from AVONET, it was present in all of our other databases meaning we were able to match it to species-level traits without using synonyms, and so we kept it in our species list without renaming. This left us with 690 of our initial 708 species.

The binomial names for three of the known host species in CLOVER could not be found in AVONET, motivating the following changes to binomial names in CLOVER based on species entries in Avibase[^2^](https://www.zotero.org/google-docs/?2QtHKl):

- Eurasian jackdaw, *Coloeus monedula*, replaced with *Corvus monedula;*
- Mongolian gull, *Larus mongolicus*, replaced with *Larus cachinnans;*
- Little cuckoo, *Piaya minuta*, replaced with *Coccycua minuta.*

We replaced the binomial names of the following four species in EltonTraits to ensure that every species from EltonTraits could be matched with a single species from eBird Status and Trends:

- Long-tailed pipit, *Anthus longicaudatus*, replaced with *Anthus vaalensis*
- Iquitos gnatcatcher, *Polioptila clementsi*, replaced with *Polioptila guianensis*
- Bluntschli’s vanga, *Hypositta perdita*, replaced with *Oxylabes madagascariensis*
- Vietnamese pheasant, *Lophura hatinhensis*, replaced with *Lophura edwardsi*
- Yellow warbler, *Setophaga petechia*, replaced with *Dendroica petechia*

In the IUCN trait data we changed the binomial name of the yellow-throated parrotbill from *Suthora webbiana* to *Sinosuthora webbiana*.

**Derivation of behavioural season boundaries**

The species metadata available through eBird Status and Trends includes fields listing the start and end dates of the breeding, nonbreeding, post-breeding migration, and pre-breeding migration seasons for species which exhibit these seasons[^6^](https://www.zotero.org/google-docs/?GoyCCP). These are listed as calendar dates during the calendar year 2021, when the data underlying the 2022 release of the Status and Trends data was collected. These dates are to a resolution of one week, so that each date occurred on a Monday and “consecutive” dates are 7 days apart. Not all species have season start and end dates listed for all four behavioural seasons, with some species having no dates listed or dates listed for a subset of the four seasons. However, we were able to directly verify that for each species with start and end dates present for at least one season, the times between the listed dates cover the entire calendar year; equivalently, the list of species with a behavioural season listed for any given day is consistent throughout the year.

Of the 708 species listed as being present in Europe in eBird Status and Trends, 546 species had behavioural season start and end dates listed. For each of these 546 species, we used the season start and end dates listed in eBird Status and Trends to assign a behavioural season for each day of the calendar year 2021. We then calculated the total number of species in each behavioural season for each day of the year to give a daily frequency distribution of species in each behavioural season. This time-varying distribution is plotted in Supplemental Figure S1A. Visual inspection shows that at the cross-species level the dominant behavioural-seasonal trends move in waves, with successive periods where the majority of species are in each of the successive behavioural seasons. To define cross-species season start and end dates, we calculated the first date for each behavioural season on which a plurality of species are in that behavioural season; that is, the first date on which the number of species in that behavioural season is larger than the number in any other season (note that since the majority of species are in the nonbreeding season at the start of calendar year 2021, we defined the start of the nonbreeding season to be the day after the last day on which a plurality of species were in the post-breeding season; visual inspection of the daily frequency distributions confirms that these two seasons do indeed follow each other at the cross-species level). The resulting behavioural season start/end dates were: non-breeding season, 30th November - 28th February (calendar days 334 - 59); pre-breeding migration, 1st March - 6th June (days 60 - 157); breeding season, 7th June - 9th August (days 158 - 221); and post-breeding migration, 10th August - 29th November (days 222 - 333). For data from leap years in our model training and testing, we considered 29th February as part of the non-breeding season.

**Random intercept models**

We attempted to augment both the basic and cross-seasonal models by specifying a random intercept according to country. We labelled each raster cell by the country its coordinates lie within using the R package rworldmap, v1.3.8[^7^](https://www.zotero.org/google-docs/?fa9IyP), and used the random intercept option provided in *embarcadero* to fit the random intercept term. For each model we ran a single MCMC chain to generate 1000 posterior samples. The random intercept term is intended to capture country-level heterogeneities in H5 HPAI detection and reporting capacity. However, since our pseudo-absence generation process accounts for spatial heterogeneity in observation processes through its weighting on citizen bird observation intensity data, there is a possibility that introducing a random intercept term may be “double accounting” for spatial heterogeneity, motivating us to compare models with and without random intercept. Along with this double accounting problem, the substantial spatial heterogeneity in our data means that the testing data for the different dataset-season combinations consistently contains data from countries which do not appear in the corresponding training data, which is likely to make assessment of model performance difficult.

Performance metrics for the models with random intercept included are listed in Supplemental Table S1. Comparison with Table 4 of the main text suggests that the random intercept models do not offer a substantial improvement in predictive ability over the corresponding models without random intercept terms, sensitivity being notably weaker for period B models.

**Bibliography for Supplemental Methods S1**

[1. Tobias, J. A. *et al.* AVONET: morphological, ecological and geographical data for all birds. *Ecol. Lett.* **25**, 581–597 (2022).](https://www.zotero.org/google-docs/?mkqb6Y)

[2. Lepage, D. Avibase - Bird Checklists of the World. *Avibase* https://avibase.bsc-eoc.org/checklist.jsp?region=EUR.](https://www.zotero.org/google-docs/?mkqb6Y)

[3. Callaghan, C. T., Nakagawa, S. & Cornwell, W. K. Global abundance estimates for 9,700 bird species. *Proc. Natl. Acad. Sci.* **118**, e2023170118 (2021).](https://www.zotero.org/google-docs/?mkqb6Y)

[4. Grey-headed Swamphen - eBird. https://ebird.org/species/purswa3.](https://www.zotero.org/google-docs/?mkqb6Y)

[5. Milvago chimachima (Yellow-headed Caracara) - Avibase. https://avibase.bsc-eoc.org/species.jsp?lang=EN&avibaseid=BA33DDD5D79EAB89.](https://www.zotero.org/google-docs/?mkqb6Y)

[6. Fink, D. *et al.* eBird Status and Trends, Data Version: 2019; Released: 2020. Cornell Lab of Ornithology, Ithaca, New York. https://doi.org/10.2173/ebirdst.2022 (2023).](https://www.zotero.org/google-docs/?mkqb6Y)

[7. South, A. rworldmap: A New R Package for Mapping Global Data. *R J.* **3**, 35–43 (2011).](https://www.zotero.org/google-docs/?mkqb6Y)

**Supplemental Table S1** Posterior means of performance metrics for random intercept models on each dataset’s test set. AUROC denotes Area Under the Receiver-Operating Characteristic curve, CV denotes covariates. Presence data in test set A is from the 2020-2021 H5N8 HPAI clade 2.3.4.4b outbreak. Presence data in test set B is from 1/3/2023 to 29/2/2024, during the ongoing (since 2021) H5N1 HPAI clade 2.3.4.4b outbreak. Values in brackets denote posterior median and 95% credible interval of metrics within individual trees. As posterior mean values are based on consensus predictions averaging over all sampled trees, this can exceed posterior medians and 95% credible intervals.

|  |  | | **Model** | |
| --- | --- | --- | --- | --- |
|  |  | | **+ random intercept** | **+ random intercept + cross-seasonal CV’s** |
|  | **nonbreeding (Nov - Feb)** | **Sens.**  **Spec.**  **AUROC** | 0.80 (0.79; 0.72-0.84)  0.82 (0.81; 0.70-0.88)  0.90 (0.87; 0.82-0.90) | 0.81 (0.79; 0.70-0.84)  0.83 (0.82; 0.75-0.87)  0.90 (0.87; 0.83-0.90) |
|  | **pre-breeding migration (Mar - Jun)** | **Sens.**  **Spec.**  **AUROC** | 0.80 (0.78; 0.70-0.85)  0.88 (0.81; 0.66-0.89)  0.91 (0.87; 0.83-0.90) | 0.75 (0.74; 0.65-0.81)  0.90 (0.88; 0.80-0.93)  0.92 (0.89; 0.85-0.91) |
| **A** | **breeding  (Jun - Aug)** | **Sens.**  **Spec.**  **AUROC** | - | - |
|  | **post-breeding migration (Aug - Nov)** | **Sens.**  **Spec.**  **AUROC** | 0.81 (0.77; 0.56-0.91)  0.86 (0.81; 0.63-0.94)  0.91 (0.88; 0.79-0.91) | 0.92 (0.89; 0.84-0.95)  0.76 (0.75; 0.65-0.80)  0.87 (0.86; 0.82-0.90) |
|  | **nonbreeding (Nov - Feb)** | **Sens.**  **Spec.**  **AUROC** | 0.80 (0.80; 0.74-0.85)  0.86 (0.86; 0.81-0.92)  0.93 (0.92; 0.89-0.94) | 0.83 (0.82; 0.77-0.88)  0.82 (0.82; 0.76-0.87)  0.92 (0.91; 0.87-0.92) |
|  | **pre-breeding migration (Mar - Jun)** | **Sens.**  **Spec.**  **AUROC** | 0.57 (0.59; 0.43-0.74)  0.80 (0.73; 0.58-0.83)  0.75 (0.72; 0.67-0.77) | 0.75 (0.75; 0.64-0.82)  0.65 (0.61; 0.52-0.74)  0.78 (0.75; 0.69-0.78) |
| **B** | **breeding  (Jun - Aug)** | **Sens.**  **Spec.**  **AUROC** | 0.78 (0.77; 0.70-0.84)  0.68 (0.67; 0.59-0.73)  0.80 (0.78; 0.76-0.81) | 0.81 (0.80; 0.75-0.86)  0.65 (0.62; 0.54-0.72)  0.80 (0.78; 0.73-0.81) |
|  | **post-breeding migration (Aug - Nov)** | **Sens.**  **Spec.**  **AUROC** | 0.76 (0.76; 0.69-0.83)  0.86 (0.77; 0.67-0.85)  0.86 (0.84; 0.79-0.87) | 0.77 (0.76; 0.69-0.82)  0.83 (0.79; 0.71-0.86)  0.88 (0.85; 0.81-0.87) |
